# Proteomic profiling in personalized nutrition: a systematic review and methodological frameworks of randomized controlled trials

**DOI:** 10.3389/fnut.2026.1826381

**Published:** 2026-06-10

**Authors:** Mirko Marino, Sofia Tura, Marco Rendine, Daniela Martini, Cristian Del Bo’, Patrizia Riso

**Affiliations:** Department of Food, Environmental and Nutritional Sciences (DeFENS), Università degli Studi di Milano, Milan, Italy

**Keywords:** biomarkers, methodological mapping, personalized nutrition, proteomics, randomized controlled trials

## Abstract

**Background:**

Personalized nutrition aims to tailor dietary advice by accounting for interindividual variability. However, the application and integration of proteomic profiling within dietary intervention studies have not yet been systematically mapped.

**Methods:**

A PRISMA-aligned systematic review (PROSPERO CRD420251011886) of randomized controlled trials (RCTs) was conducted searching PubMed, Embase, and Scopus. We included RCTs with a dietary intervention incorporating a genotype- and/or phenotype-based personalization component and protein profiling measured with high-throughput or multiplex platforms (including MS-based, aptamer-based, and multiplex immunoassays).

**Results:**

Twenty-six trials were included, with sample sizes ranging from 14 to 805 participants. Most interventions were supplementation-based (*n* = 19), whereas seven trials assessed food-based interventions. Personalization strategies were mostly phenotype-based (*n* = 17), less frequently genotype-based (*n* = 7), and only one trial implemented a fully individualized dietary intervention. Proteomic analyses were mainly performed on blood-derived matrices (*n* = 24). Targeted profiling approaches predominated (*n* = 20), most commonly using multiplex immunoassays, while untargeted mass spectrometry-based methods were less frequent (*n* = 6). Due to substantial heterogeneity in study designs, analytical platforms, and outcome definitions, a quantitative synthesis was not feasible. Most trials were judged at high risk of bias, mainly driven by missing outcome data and selective reporting, particularly in exploratory and subgroup analyses.

**Conclusion:**

In personalized nutrition RCTs, proteomic profiling is currently employed predominantly as a targeted, secondary outcome to characterize subgroup-specific molecular responses, with limited use as a tool to inform stratification or guide intervention design. Future research should prioritize adequately powered trials with pre-specified proteomic outcomes and the integration of standardized proteomic workflows to enhance interpretability and clinical translation.

## 1 Introduction

Traditional nutritional guidelines and recommendations have been developed at the population level, with the primary objectives of preventing nutrient deficiencies and reducing the burden of chronic diseases. Although this approach has yielded substantial public health benefits, it assumes largely homogeneous responses and does not fully account for interindividual differences in genetics, physiology, environment, and behavior (1, 2). In this context, the field of personalized nutrition (PN) has emerged, aiming to tailor dietary interventions to the individual level (3). PN encompasses approaches that differ in the level of individualization. Stratified nutrition refers to recommendations targeted to subgroups sharing specific characteristics, whereas tailored nutrition adapts dietary advice based on selected individual features. Precision nutrition generally implies a more comprehensive integration of multi-dimensional data, while personalized nutrition represents the most individualized level, aiming to integrate genetic, phenotypic, clinical, lifestyle, and environmental information to optimize health outcomes (3–6). While stratified or tailored nutrition provides recommendations for subgroups sharing specific characteristics, PN extends this approach to the individual by integrating genetic, phenotypic, clinical, lifestyle, and environmental information to optimize health outcomes (4, 6). The biological basis of PN lies in the interplay of genotype and phenotype. Genetic variability, captured through single nucleotide polymorphisms (SNPs) or aggregated into genetic risk scores (GRS), influences nutrient metabolism, dietary responsiveness, and disease susceptibility (2, 7–13). The phenotype, including both clinical features (age, sex, body mass index, body composition, health status) and molecular traits (metabolites, protein expression, microbiome composition), reflects dynamic interactions between genetic predisposition and environmental exposures and modulates dietary responses (14–19). Behavioral and environmental factors further contribute to shaping these responses (3, 20, 21).

Omics technologies (i.e., genomics, transcriptomics, proteomics, and metabolomics) enable the characterization of molecular mechanisms underlying individual responses to diet. Nutriomics, defined as the integration of nutrition research with omics platforms, facilitates the identification of individual molecular signatures of dietary exposure (4, 22–27). When combined with machine learning and high-throughput data acquisition, multi-omics approaches can manage and interpret large-scale datasets, facilitating the identification of metabolic subtypes and predictive markers that may inform personalized dietary recommendations (24–27). Among these technologies, proteomics offers a direct insight into functional molecular responses, as proteins represent the primary effectors of biological processes. Unlike static genomic information, the proteome is dynamic and responsive to dietary and environmental stimuli, reflecting changes in protein expression, post-translational modification and molecular interactions (28–30). Proteomics has evolved from low-throughput biochemical techniques to high-throughput platforms, including mass spectrometry and other multiplex and affinity-based assays (31–36). These technologies allow comprehensive profiling of proteins in various biological matrices, providing both qualitative and quantitative profiling of proteins across diverse biological matrices, thereby facilitating the elucidation of molecular mechanisms linking dietary exposure to health outcomes (37–41). Nutritional proteomics, or nutriproteomics, applies these tools to investigate diet–protein interactions, contributing to biomarker discovery and potentially supporting the design of personalized nutrition strategies guided by individual proteomic profiles (39–41). Despite these technological advancements, the integration of high-throughput proteomics (HTP) into PN interventions remains relatively unexplored in randomized controlled trials (RCTs). Existing studies and narrative or omics-focused reviews have primarily examined associations between diet and proteomic profiles or discussed technological potential, with limited attention to how proteomic data are operationally incorporated into intervention design and implementation. To date, no systematic synthesis has examined how proteomic profiling is operationally embedded in personalized nutrition RCTs, whether it serves as a driver of intervention design or is primarily employed as a secondary outcome measure, and how these differing applications influence methodological rigor and translation readiness.

The present systematic review provides a methodological mapping of the use of proteomic profiling in personalized nutrition RCTs. Specifically, it summarizes how proteomics is operationalized in intervention design and evaluation, including whether it informs personalization strategies or is primarily used as an outcome measure. By systematically characterizing these aspects, this review aims to identify current methodological limitations and to inform the design of future proteomics-driven personalized nutrition trials.

## 2 Methods

This systematic review was conducted in accordance with the PRISMA (Preferred Reporting Items for Systematic Reviews and Meta-Analyses) guidelines (42) and was prospectively registered in PROSPERO (CRD420251011886).

### 2.1 Eligibility criteria

Eligibility criteria were defined using the PICOS framework (43), which guided the formulation of the research question and supported the development of a structured search strategy. The PICOS elements are summarized in Table 1. Given the evolving definition of personalized nutrition, dietary intervention studies were considered eligible when personalization was implemented either: (i) *a priori*, as individualized dietary interventions tailored to participant characteristics, or studies enrolling populations selected based on a specific genotype, even if all participants received the same intervention; (ii) a posteriori, when all participants received the same intervention, but outcomes were analyzed according to stratification by genotypic or phenotypic characteristics, assessed at baseline or emerging post-intervention.

**Table 1:** PICOS framework applied to the present review.

PICOS element	Eligibility criteria
Population (P)	Children, adolescents, adults and older subjects, regardless of anthropometric and health/pathological status, hospitalized or not.
Intervention (I)	Dietary interventions in which personalization was applied, either by personalizing the intervention to individual characteristics (e.g., genotypic or phenotypic profiles), by conducting the study in populations defined by specific genotypes, or by stratifying outcomes according to baseline or post-intervention genotypic/phenotypic features.
Comparison (C)	Control group (placebo, standard/control diet) or comparison between different subgroups.
Outcome (O)	Protein changes assessed via high-throughput proteomic analysis (e.g., mass spectrometry, protein pathway array, Olink, multiplex bead-based assays, multiplex aptamer-based assays, proteomic chip).
Study design (S)	Randomized controlled trials.

#### 2.1.1 Exclusion criteria

Studies were excluded if they: (i) were conducted exclusively in athlete populations; (ii) did not report intervention-related changes in specific proteins or protein panels (i.e., proteomic outcomes were not presented at the protein level); or (iii) were not published in English.

#### 2.1.2 Justification for genotype- and phenotype-based inclusion

Genotype-selected studies were included even when the interventions were identical across participants, as genotypic variation is inherent and stable, enabling the evaluation of differential responses attributable to genetic differences. In contrast, phenotype-selected studies were included only if the study design enabled meaningful comparisons across phenotypic strata. Recruitment based solely on phenotypic characteristics, without the capacity to assess differential outcomes, was considered insufficient for inclusion. Athlete-focused studies were excluded because dietary interventions in these populations are typically designed to address sport-specific performance goal rather than individualized health-related nutritional requirements.

#### 2.1.3 Proteomic inclusion criteria

Studies were considered to use high-throughput proteomics if protein measurement was conducted using platforms such as mass spectrometry, protein arrays, or multiplex-based technologies, irrespective of the number of proteins quantified; no minimum threshold was applied. Proteomic approaches were categorized as targeted or untargeted according to the analytical approach described. Studies were excluded if they did not report which proteins or protein components were modulated by the intervention.

#### 2.1.4 Study design and publication criteria

Only RCTs were included to ensure a high level of evidence for intervention effects. Observational studies, case reports, study protocols, and other non-randomized designs were excluded to reduce the risk of confounding and enhance methodological comparability across included studies. We acknowledge that observational studies are common in the field of personalized nutrition and can provide valuable insights into real-world associations and hypothesis generation; however, they were excluded in order to focus on intervention-based evidence and to improve the internal validity and comparability of proteomic outcomes across studies.

Eligibility was further restricted to articles published in English between January 2010 and May 2025 to capture recent developments in personalized nutrition and high-throughput proteomic profiling.

### 2.2 Search strategy

A comprehensive literature search was conducted in PubMed, Embase, and Scopus, to identify papers published from January 2010 to 25 May 2025. Search filters were applied to restrict results to human studies published in English during the specified time frame. The initial search yielded 5,447 records. The complete search strings for each database are reported in Supplementary material 1 (Research strategy).

### 2.3 Study selection

To ensure reliability, all records were independently screened and selected by two reviewers (S.T. and M.R.), with discrepancies resolved by a third reviewer (M.M.). Retrieved records were imported into Rayyan for deduplication and screening. After removal of duplicates, title and abstract were screened according to predefined inclusion and exclusion criteria. The remaining records underwent full-text assessment to confirm eligibility. Full-text screening was performed manually. Any uncertainty regarding study inclusion was resolved through discussion among two reviewers (S.T. and M.R.), with the consultation of a third reviewer (M.M.) in case of disagreement.

### 2.4 Data extraction

Data were independently extracted by two investigators (S.T. and M.R.) using a standardized Excel spreadsheet to ensure consistency across studies. For each included trial, general information was first recorded, including the article title, year of publication, and country of origin. Additional information included population characteristics (sample size, age, sex distribution, body mass index, and baseline health status), study design (duration of the intervention, type of intervention, dietary vs. supplementation), comparator condition (e.g., standard diet, placebo, or other control). Furthermore, the personalization strategy adopted in each study was classified as either phenotype-based or genotype-based. Regarding the proteomics component, the analytical platform used (e.g., mass spectrometry, protein pathway arrays, multiplex-based methods, or Olink) was recorded, along with whether the approach was targeted or untargeted. Finally, the biological matrix analyzed (e.g., plasma, serum, urine, or other sample types) and the proteomic outcomes assessed (i.e., specific proteins or protein panels) were documented.

### 2.5 Risk of bias assessment

The risk of bias of the included RCTs was independently assessed by two reviewers (S.T. and M.R.) using the Cochrane Risk of Bias 2 (RoB-2) tool (44). Any disagreements were resolved through discussion between the two reviewers, and when consensus was not reached, a third reviewer (M.M.) was consulted for adjudication.

### 2.6 Data synthesis

Given the heterogeneity of interventions, personalization frameworks, platforms, and outcome definitions, we did not perform meta-analysis. Results were synthesized descriptively. The synthesis was structured according to the type of personalization strategy (genotype-based, phenotype-based, combined) and the proteomic approach employed (targeted vs. untargeted). Emphasis was placed on identifying reporting patterns and key methodological indicators including study type, trial registration, choice of analytical platform, and risk-of-bias domains, particularly those related to missing outcome data and selective reporting.

## 3 Results

### 3.1 Study selection

**Figure 1:**
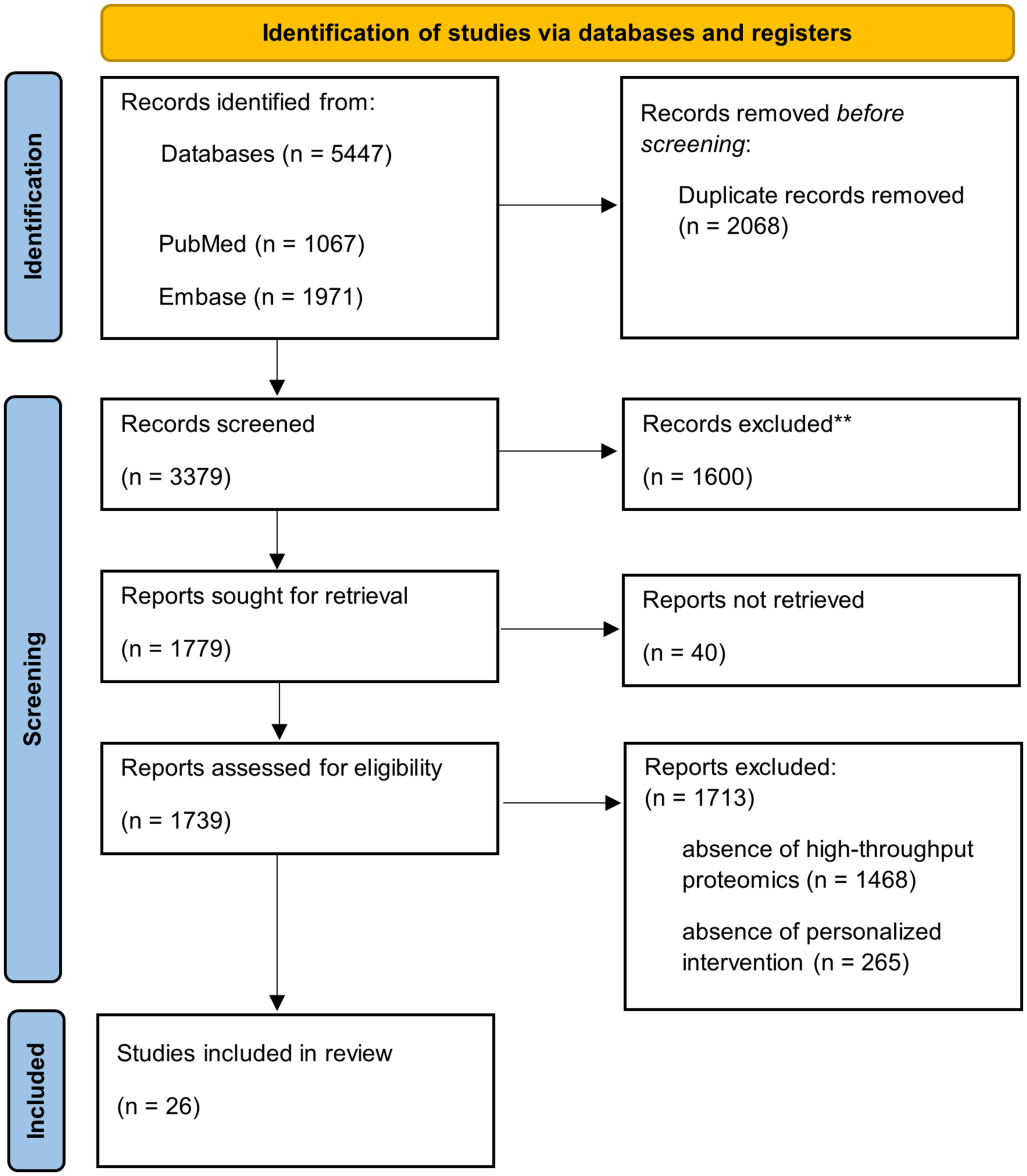
PRISMA flow diagram of the systematic review.

The study selection process was conducted and reported in accordance with the PRISMA 2020 guidelines. The selection workflow is summarized in the PRISMA flow diagram (Figure 1). A total of 5,447 records were identified through searches of three electronic databases. After removal of 2,068 duplicates, 3,379 records were screened by title and abstract, leading to the exclusion of 1,600 articles. Of the 1,779 full texts assessed for eligibility, 40 could not be retrieved and 1,713 were excluded for reasons including lack of high-throughput proteomic analyses, absence of a dietary intervention or personalization strategy, non-randomized design, or non-English publication. Reasons for exclusion were not mutually exclusive. Therefore, the sum of exclusions by reason exceeds the total number of excluded reports. Ultimately, 26 RCTs were included in the systematic review (45–70).

### 3.2 General characteristics of included studies

The main characteristics of the included trials are summarized in Supplementary material 2 (Data extraction). The studies were published between 2011 and 2025 and included diverse geographical settings, including United States (*n* = 5) (49, 52, 54, 61, 68), Spain (*n* = 3) (56, 64, 67), Norway (*n* = 2) (46, 51). Of the 26 trials, 11 reported primary RCT findings (49, 53–55, 58, 60, 62, 65, 66, 70, 71), whereas 15 were secondary analyses of previously conducted RCTs (45–47, 49–51, 55, 56, 58, 60, 62, 63, 66–68). Trial registration was reported in 19 studies (45–47, 49–63, 67). Nineteen studies (46–49, 53–65, 67, 70) employed parallel-group design, while 7 used a crossover design (45, 50–52, 66, 68, 69). The nutritional interventions were heterogeneous and predominantly supplementation-based (*n* = 19) (45–47, 50–55, 57–60, 62, 63, 65, 66, 69, 70), including *ω*-3 fatty acids, probiotics, micronutrients, and bioactive compounds. The remaining 7 trials assessed dietary or food-based interventions (48, 49, 56, 61, 64, 67, 68), such as macronutrient modifications, specific dietary patterns, or individualized dietary approaches. Sample sizes varied considerably ranging from 14 to 805 participants, and intervention duration ranged from 4 weeks to 36 months. Four studies were conducted in pediatric population (58–60, 63), whereas the remaining trials enrolled adults, including sex-specific cohorts. Study populations ranged from healthy individuals to participants with metabolic or clinical conditions including obesity, type 2 diabetes, coronary heart disease, cirrhosis, anemia, and allergy. Five studies selected participants based on specific genotypic characteristics (50, 51, 53, 59, 63), including familial hypercholesterolemia, β^0^-thalassemia/Hb E, Duchenne muscular dystrophy–related deletions, or genetic susceptibility to type 1 diabetes. Personalization strategies were implemented through genotype- or phenotype-based stratification in all trials, although only one study applied a fully individualized dietary intervention. Proteomic analyses were primarily conducted in blood-based matrices (*n* = 24) (45–53, 55–59, 61–70), with only two studies analyzing alternative biological matrices (nasal fluid and fecal samples) (54, 60).

### 3.3 Personalization approaches

All trials included incorporated at least one level of personalization, in accordance with the predefined eligibility criteria. Personalization strategies were heterogeneous, differing both in their biological basis (phenotype-based, genotype-based, or combined) and in the timing of their implementation, either at baseline or following the dietary intervention. Phenotype-based personalization was the most frequently adopted approach, reported in 17 studies (45–49, 52, 56–58, 60–62, 65–67, 69, 70). In 8 trials (47, 48, 57, 58, 60, 61, 65, 66), personalization was implemented at baseline through stratification according to individual characteristics, including metabolic, biochemical, physiological, or clinical features. The criteria used for baseline stratification varied considerably, reflecting diverse conceptual frameworks of personalization. In 9 studies phenotype-based personalization was applied through exploratory analyses, in which participants were stratified after completion of the intervention (45, 46, 49, 52, 56, 62, 67, 69, 70). Among these, 5 studies (46, 49, 52, 62, 67) applied phenotype-based personalization post-intervention stratifying participants according to differential responses to a common dietary intervention. In most cases, participants were categorized as responders or non-responders based on changes in clinical or biological outcomes, while one study classified participants according to weight maintenance success. Genotype-based personalization was applied in 7 studies. In all cases (50, 51, 54, 59, 63, 64, 68), genetic characteristics were used *a priori* to define the study population or stratify participants at baseline. Although in a minority of cases some participants were classified based on phenotypic or familial criteria rather than direct genotyping, the personalization strategy was primarily driven by genetic determinants. Two studies combined multiple personalization approaches within the same trial (53, 55). These included combinations of genotype-based recruitment alongside phenotype-based stratification, as well as integration of both baseline and post-intervention phenotype-based personalization. In one study (53), genotype was used as an inclusion criterion at baseline and phenotype stratification was conducted as an exploratory analysis. In the other study (55) both genotype- and phenotype-based personalization were investigated through post-intervention exploratory analyses. Only one study implemented a fully individualized dietary intervention, tailoring nutritional recommendations according to individual leukocyte activation test results (61), representing an extreme form of phenotype-based personalization at the individual level. This study was classified within the baseline stratification group. Overall, the included studies demonstrated substantial heterogeneity in personalization strategies, both in terms of biological basis and timing of application.

### 3.4 Proteomic approaches and investigated pathways

Across the included studies, proteomic analyses were predominantly based on targeted approaches. Twenty trials employed targeted proteomics and 6 adopted untargeted strategies. In targeted studies, the number of proteins assessed varied widely, ranging from single biomarkers to large multiplex panels comprising more than 1,000 proteins (48, 61, 62). Only one study conducted a metaproteomic analysis focused on microbial protein profiling (61). Targeted proteomic profiling was most frequently performed using multiplex immunoassays (*n* = 17), representing the predominant analytical approach across the included trials (45–50, 53–55, 57–59, 63, 64, 66, 69, 70). These platforms were primarily based on bead-based or electrochemiluminescence technologies. MS-based proteomics constituted the second most frequently adopted strategy (*n* = 9) (45, 51, 52, 56, 60, 62, 65, 67, 68) and exhibited considerable heterogeneity in analytical depth, encompassing both targeted and discovery-driven workflows. An aptamer-based assay was used in one study (61). The proteomic targets were mainly circulating proteins involved in inflammation, metabolic regulation, and iron metabolism, reflecting the predominant use of blood-derived matrices (i.e., plasma and serum). Inflammatory pathways represented the most frequently investigated biological domain. Cytokines were the most assessed markers and were often analyzed in combination with other inflammatory mediators, including acute-phase proteins, chemokines, growth factors, and adhesion molecules. Adipokines were evaluated in a smaller number of studies, either alone or in combination with inflammatory markers. Proteins related to iron metabolism were investigated in studies focusing on iron supplementation, while lipid metabolism–related proteins were assessed in a single trial involving participants with familial hypercholesterolemia. Finally, six studies (52, 56, 60, 65, 67, 68) adopted exploratory or discovery-based proteomic strategies without predefining specific protein targets. Overall, the included trials primarily relied on targeted multiplex approaches, complemented by a smaller number of untargeted analyses aimed at identifying broader proteomic signatures.

### 3.5 Proteomic findings and outcomes

Across the included studies, proteomic outcomes were reported using heterogeneous platforms, protein panels, and outcome definitions. Accordingly, quantitative synthesis was not feasible, and results are summarized descriptively by the main personalization strategy.

#### 3.5.1 Genotype-based personalization

Studies applying genotype-based personalization (*n* = 7) (50, 51, 54, 59, 63, 64, 68) primarily investigated supplementation-based interventions and focused on targeted assessments of inflammatory or metabolic protein profiles. Most trials evaluated cytokine responses following *ω*-3 or DHA supplementation, while a smaller number of studies investigated adipokines, lipid metabolism proteins, or employed untargeted proteomic approaches. Overall, these studies suggested that genetic background modulates inflammatory and metabolic proteomic responses to dietary interventions. Genotype-specific differences were reported in cytokine regulation, apolipoprotein dynamics, and broader proteomic signatures. However, variability in the specific protein targets assessed and in reporting formats across trials limited direct comparability of findings.

#### 3.5.2 Phenotype-based personalization

Studies adopting phenotype-based personalization (*n* = 17) (45–49, 52, 56–58, 60–62, 65–67, 69, 70) assessed proteomic responses according to clinical, biochemical, or physiological characteristics, stratifying at baseline prior to the intervention (*n* = 8) (47, 48, 57, 58, 60, 61, 65, 66), or stratifying participants post-intervention through exploratory analyses (*n* = 9) (45, 46, 49, 52, 56, 62, 67, 69, 70). Phenotype-based studies predominantly used targeted multiplex platforms to investigate inflammatory markers, adipokines, and, less frequently, iron metabolism proteins. Across these trials, differential regulation of inflammatory cytokines, adhesion molecules, adipokines, or iron-related proteins was frequently observed according to baseline phenotype, suggesting that initial physiological status influenced molecular responses to dietary interventions. Untargeted proteomic analyses conducted within phenotype-based frameworks revealed broader pathway-level differences, including modulation of immune response, lipid metabolism, coagulation, and microbial metabolic activity. These exploratory approaches highlighted phenotype-dependent proteomic signatures and, in some cases, suggested that dietary interventions attenuated baseline-related molecular differences. Post-intervention phenotype-based stratification further demonstrated distinct proteomic patterns between responders and non-responders across heterogeneous interventions (46, 49, 52, 62, 67), including supplementation and dietary strategies. In these studies, both targeted and untargeted approaches identified response-specific molecular profiles involving inflammatory mediators, lipid metabolism, immune response, and complement pathways. Reporting was generally study-specific and frequently embedded within subgroup analyses, limiting cross-study comparability.

#### 3.5.3 Multiple personalization approaches

Two studies combined genotype- and phenotype-based strategies or applied multiple phenotype-based classifications within the same trial (53, 55). These studies generally employed targeted proteomic profiling and reported differential molecular responses according to combined stratification criteria. Overall, heterogeneity in personalization frameworks, platforms, and outcome reporting limited the possibility of cross-study synthesis beyond descriptive mapping.

#### 3.5.4 Comparative synthesis of personalization approaches

**Figure 2:**
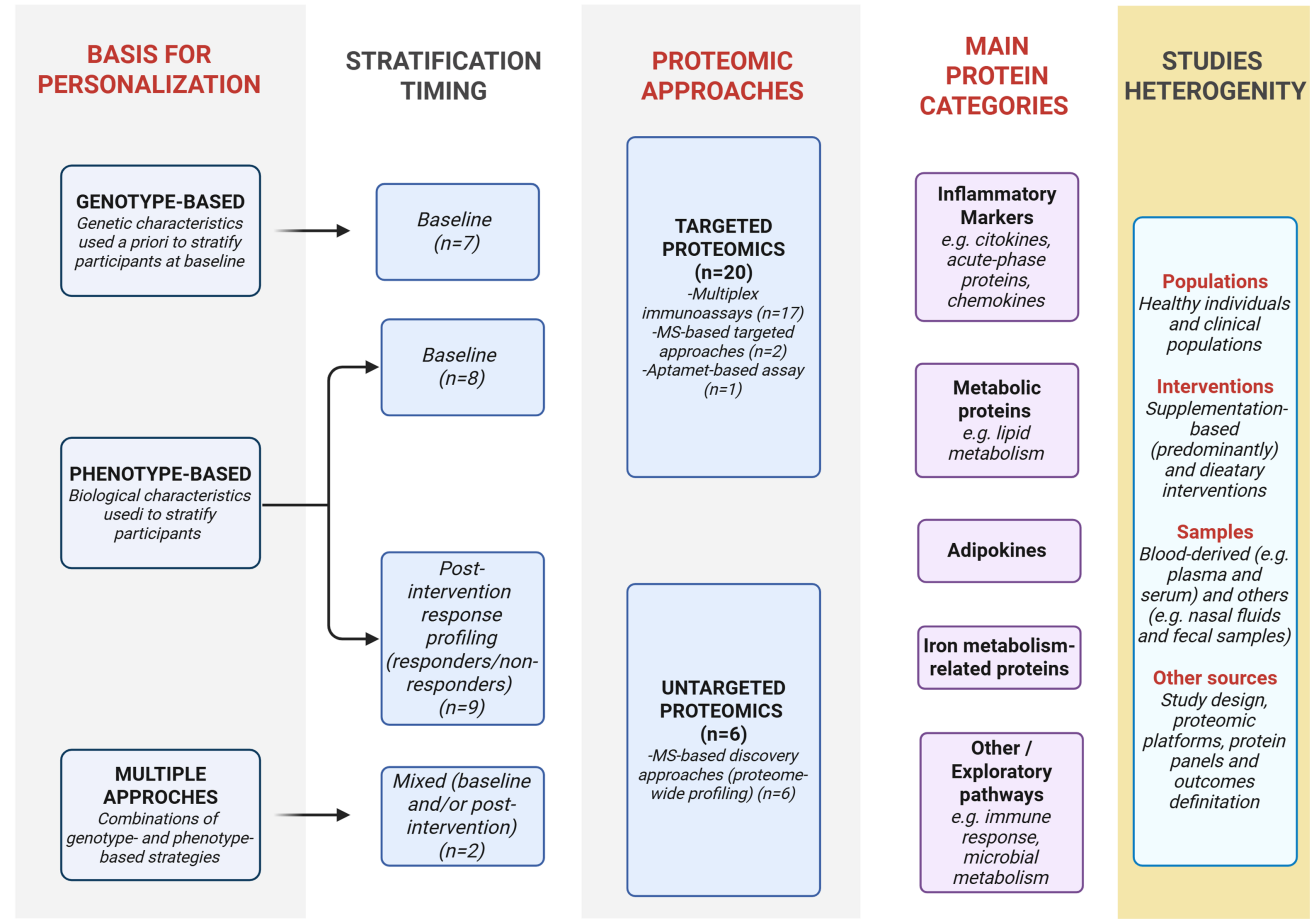
Overview of personalization approaches and proteomic strategies across included studies. Stratification timing is categorized as baseline (pre-specified) or post-intervention (exploratory). Counts refer to RCTs. Each RCT was assigned to a single primary category for personalization approach and proteomic strategy.

An overview of the distribution of personalization approaches, stratification timing, and proteomic strategies across the included studies is presented in Figure 2. Genotype-based studies generally used genetic characteristics *a priori*, either to define the study population or to stratify participants at baseline, and most often assessed predefined inflammatory or metabolic protein profiles, particularly cytokines and lipid-related proteins. In contrast, phenotype-based studies showed greater methodological variability, including baseline stratification based on clinical or physiological characteristics and post-intervention responder/non-responder classifications. Across both approaches, inflammatory and metabolic protein panels were the most commonly investigated domains, but the specific proteins assessed, analytical depth, and reporting formats differed substantially across studies. Overall, genotype-based approaches were more closely linked to predefined biological characteristics and protein panels, whereas phenotype-based approaches captured a broader range of stratification criteria but were also more heterogeneous.

### 3.6 Risk of bias assessment

**Figure 3:**
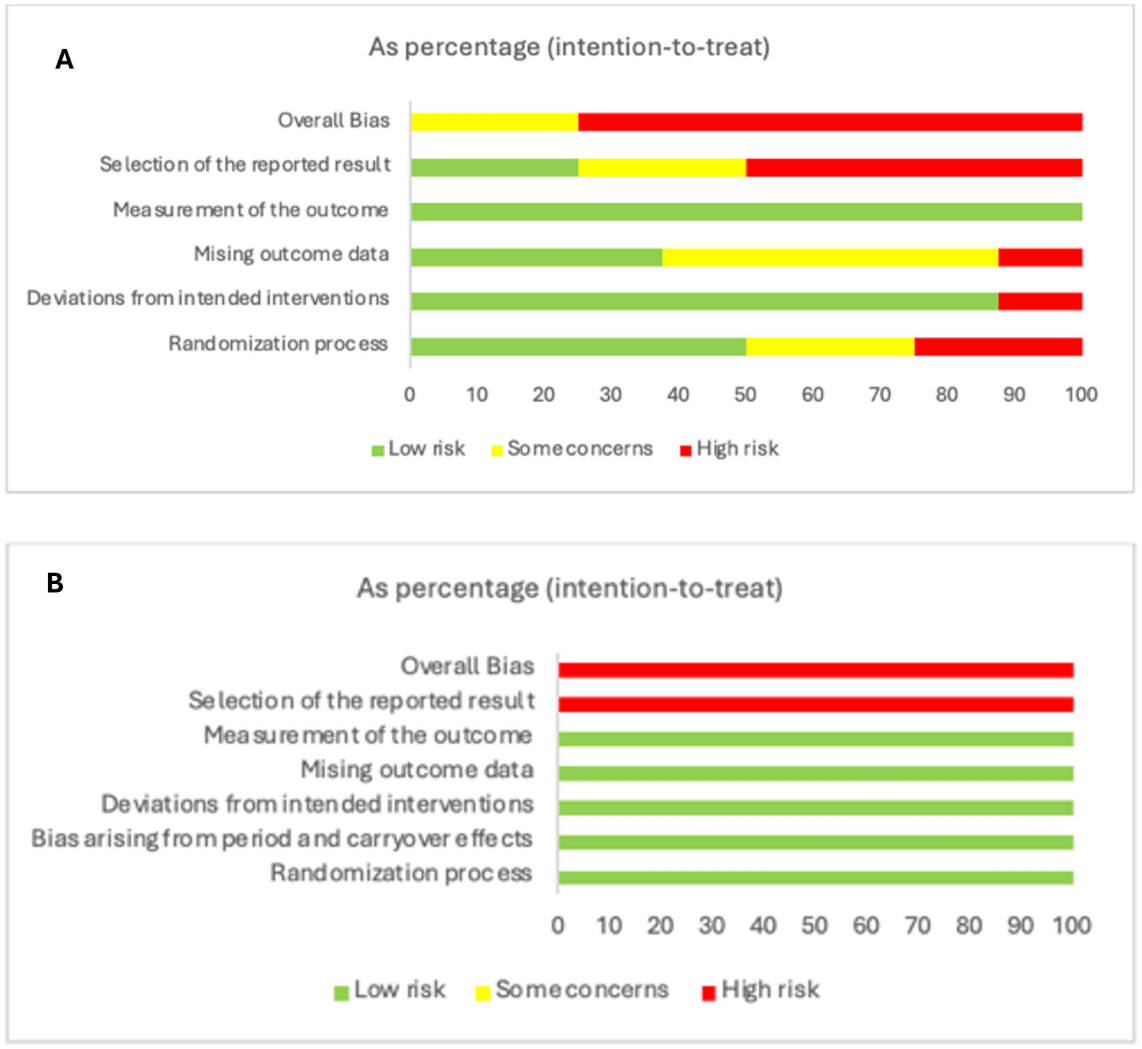
Summary of the risk-of-bias assessment expressed as percentages according to the ITT principle for parallel trials **(A)** and for crossover trials **(B)**.

**Figure 4:**
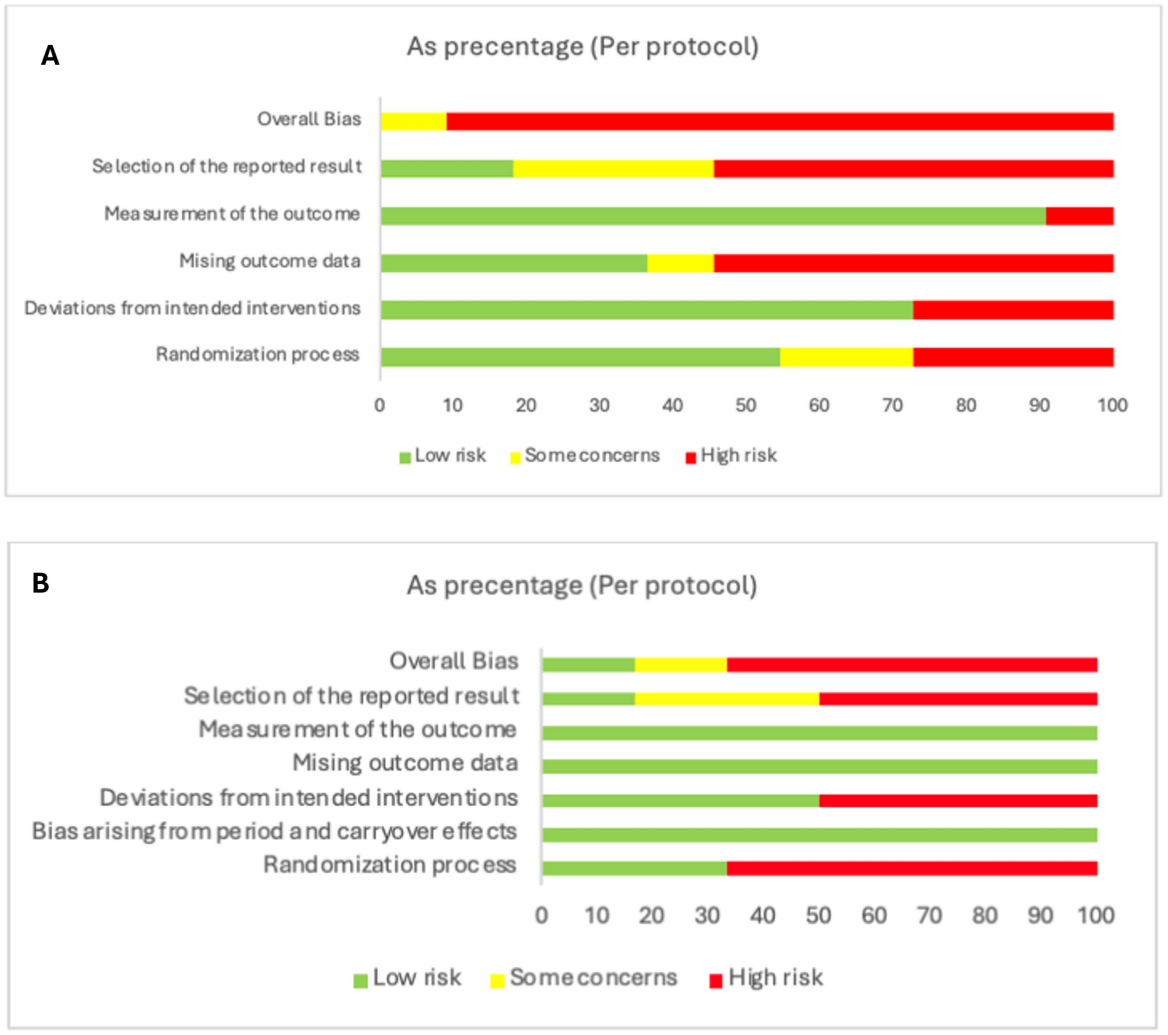
Summary of the risk-of-bias assessment expressed as percentages according to the PP principle for parallel trials **(A)** and for crossover trials **(B)**.

The risk of bias of the 26 included randomized controlled trials was assessed using the Cochrane Risk of Bias 2 (RoB 2) tool, applying design-specific algorithms for parallel-group and crossover trials and considering both intention-to-treat (ITT) and per-protocol (PP) analyses where available. Overall, most studies were judged to have a high risk of bias. In parallel-group RCTs, the lowest risk of bias was observed for deviations from intended interventions (D2) and outcome measurement (D4), reflecting objective assay readouts. Intermediate risk was seen for the randomization process (D1), mainly due to incomplete reporting and post-hoc subgroup analyses, while the highest risk was noted for missing outcome data (D3) and selection of reported results (D5), particularly in exploratory analyses or outcomes not pre-specified. Crossover RCTs showed similar patterns. Lowest risk was generally observed for missing outcome data (D3), outcome measurement (D4), and bias due to period or carryover effects. Higher risk was common for randomization (D1), deviations from intended interventions (D2), and selection of reported results (D5), often due to incomplete reporting, subgroup analyses, or analyses restricted to completers. Summary assessments of risk of bias according to the ITT principle are presented in Figure 3 (parallel trials, A; crossover trials, B), while corresponding PP analyses are shown in Figure 4 (parallel trials, A; crossover trials, B). Detailed domain-level assessments for each study are provided in Supplementary Figures 1, 2. Overall, although intervention delivery and objective outcome measurement were generally considered adequate, most included studies, particularly those relying on subgroup or exploratory proteomic analyses, were judged to be at high risk of bias. This assessment was primarily driven by concerns related to missing outcome data and selective outcome reporting.

## 4 Discussion

This systematic review provides a methodological mapping of RCTs that integrated protein profiling within PN interventions. The added value of this work lies in the development of a reproducible, study-level dataset that systematically quantifies how personalization strategies are implemented and how protein-profiling platforms are applied in RCT settings. The available evidence shows substantial heterogeneity across interventions, populations, personalization frameworks, and proteomic methodologies, precluding quantitative synthesis. Rather than estimating clinical effectiveness, the included studies primarily illustrate how protein profiling is currently operationalized in PN research and where methodological readiness remains limited.

Across the included trials, proteomic profiling was most frequently positioned as an ancillary or secondary endpoint, often within secondary analyses. In most cases, it was used to characterize subgroup-specific molecular responses rather than to drive stratification or intervention logic. This pattern is consistent with the observation that fully individualized PN strategies were rare, whereas most trials relied on baseline phenotype stratification or post-intervention responder classifications. As a result, the predominant role of proteomics in this body of literature appears to be descriptive (characterizing interindividual variation) rather than operational (guiding allocation, dose, or decision rules). Platform selection further reinforces this interpretation. Targeted multiplex panels predominated, largely focusing on inflammatory mediators, while untargeted MS approaches were less frequently adopted. The predominance of targeted assays likely reflects pragmatic considerations including cost, throughput, sample volume, and established workflows. However, this emphasis also constrains discovery potential and reinforces reliance on predefined candidate pathways. As a consequence, cross-study comparability is limited, given the heterogeneity in protein panels, outcome definitions, and reporting formats, as well as the frequent reliance on study-specific subgroup analyses. Collectively, the mapped literature suggests that proteomic technologies are increasingly incorporated into PN RCTs, they are still seldom embedded as a core component of trial design. Taken together, these findings indicate that the current integration of proteomics in PN remains largely exploratory and has not yet translated into decision-making frameworks within trial design.

When examined over time, the included literature suggests a shift from early exploratory MS-based profiling toward a predominance of targeted multiplex panels in more recent years. This pattern may reflect a shift from broad discovery to monitoring predefined proteins, but it should be interpreted with caution, as publication type (primary vs. secondary analyses), study aims, and platform availability differed across time periods. If confirmed in future updates of the evidence base, this trend could indicate that proteomic integration within PN research has progressed toward feasibility and standardization, albeit potentially at the expense of opportunities for discovering novel, stratification-ready molecular signatures. In particular, cytokine-focused targeted multiplex panels have become the commonly adopted approach, likely due to practical advantages such as high throughput, reproducibility, and limited sample volume requirements (33). However, most proteomic analyses were conducted as secondary or ancillary evaluations, confirming expected biological effects rather than guiding stratification or personalized intervention. Exceptions include the SomaScan trial, which applied high-coverage aptamer-based proteomics in a fully individualized intervention framework (61), and a study using hepcidin to stratify female blood donors in response to iron supplementation (62). Early exploratory studies also illustrated the potential of untargeted proteomics to identify predictive molecular signatures, such as GSTM1-dependent peptidome changes following vegetable supplementation (68) or protein profiles distinguishing successful from unsuccessful weight maintenance (67). These studies suggest an early transition from descriptive profiling toward predictive applications, although such approaches remain limited. In PN trials, the predominance of targeted proteomics reinforces a model in which molecular data are primarily used to interrogate predefined biological pathways rather than to generate novel stratification frameworks. In the majority of trials, targeted panels were used to test predefined pathways (often inflammatory and metabolic), while fewer studies used untargeted workflows intended for broader signature discovery. This aligns with broader trends in clinical research, where multiplex immunoassays are widely employed to monitor systemic inflammatory mediators (71–73). The predominance of targeted assays also reflects limitations in available disease- or phenotype-specific proteomic markers suitable for guiding stratification (74). While the SomaScan study marked a conceptual step forward in integrating large-scale proteomic data for individualized interventions, in most trials, proteomics served as a confirmatory tool, highlighting the gap between technological capability and conceptual application. Notably, the hepcidin study (62) represents one of the earliest examples of using proteomic data to stratify participants, suggesting that selected proteins could function both as biomarkers and as stratification criteria.

Although less frequently applied, untargeted proteomics enabled a broader and more exploratory assessment of proteome-wide responses. MS-based approaches enabled broader, discovery-oriented assessment of proteome-wide responses. Across heterogeneous interventions and populations, these findings suggest shared homeostatic mechanisms underlying nutritional responses, reinforcing the concept of tightly integrated metabolic–immune networks (75, 76). Sample sizes were generally small (<30 participants per group) in these studies, reflecting their exploratory objectives and challenges in standardizing analytical pipelines (77). Most studies used plasma or serum, limiting the insight into tissue-specific mechanisms, whereas the only metaproteomic study in infants provided a functional perspective on gut microbiome interactions with dietary supplementation (60, 78, 79). Validation of untargeted findings was limited, with only two early studies performing confirmatory western blot analyses (67, 68), highlighting the need for integrated discovery-validation workflows in PN research.

From a personalization standpoint, the reviewed studies confirm that proteomic responses vary according to genotype or phenotype, providing empirical support for the core PN principle that individuals may respond differently to the same dietary intervention (3, 80). However, most personalization approaches remain subgroup-based, not fully individualized. This transitional stage represents a critical phase, laying the groundwork for future studies that can combine molecular profiling with dietary intervention to achieve truly PN strategies (74). The wide heterogeneity of study populations, ranging from infants to older adults and including both healthy individuals and clinical cohorts underscores the broad applicability of PN and suggests their potential relevance across life stages and health conditions. The predominance of supplementation-based interventions, especially those targeting probiotics, micronutrients, or bioactive compounds, reflect a research focus on identifying molecular mediators capable of modulating inflammatory and metabolic homeostasis (36).

Regarding study quality, the majority of RCTs were judged at high risk of bias, consistent across both parallel and crossover designs. This was largely due to exploratory study designs, secondary analyses conducted on subsets of participants, incomplete outcomes reporting, and deviations from intention-to-treat principles (81). Outcome measurement was frequently rated at relatively low risk of bias, reflecting the objective nature of proteomic assay readouts. However, overall risk of bias judgments remained high, primarily driven by concerns related to missing outcome data and selective reporting, particularly in exploratory and subgroup analyses.

Looking ahead, the advancement of PN toward clinical implementation will depend on well-powered RCTs characterized by standardized protocols, integrated proteomic pipelines, and validation of protein biomarkers as reliable indicators of dietary responsiveness (40, 77). In this context, the standardization of proteomic workflows and the validation of clinically actionable biomarkers represent key prerequisites for translation into practice and guideline development. Expanding proteome coverage, integrating proteomics with other omics layers, and leveraging advanced computational tools, including artificial intelligence and machine learning, will be essential to enable predictive, pathway-based personalization (4, 24). At the same time, ethical and practical considerations, including data privacy, equitable access, and user-centered design, will be crucial to translating molecular insights into actionable dietary recommendations (25, 82). Proteomics is poised to play a pivotal role in elucidating the molecular mechanisms underlying interindividual variability, moving PN from descriptive observations toward mechanistic and predictive applications. The evidence reviewed here represents an early but necessary stage in this trajectory, highlighting both the promise and current limitations associated with integrating HTP approaches into nutritional research.

## 5 Conclusion

Proteomic profiling is increasingly embedded in PN RCTs. However, it is still used mainly as a targeted, secondary outcome rather than as a tool to inform stratification strategies or guide intervention decisions. Methodological readiness remains constrained by limitations in prespecification and reporting, which undermine reproducibility and cross-study synthesis.

Future research should prioritize the prespecification of proteomic endpoints and appropriate multiplicity control, ensure comprehensive reporting of results, including null findings, and adopt transparent, standardized analytical workflows incorporating predefined discovery and validation phases. Such measures are essential to enhance methodological rigor and enable scalable translation.

In addition, the high analytical costs generally associated with proteomic technologies currently limit their scalability in large-scale research settings. Progress in the field will therefore depend on securing adequate financial support and/or developing more cost-effective analytical strategies.

## References

[1] Berry SE Valdes AM Drew DA Asnicar F Mazidi M Wolf J . Human postprandial responses to food and potential for precision nutrition. Nat Med. (2020) 26:964–73. doi: 10.1038/s41591-020-0934-0, 32528151 PMC8265154

[2] Mullins VA Bresette W Johnstone L Hallmark B Chilton FH. Genomics in personalized nutrition: can you “eat for your genes”? Nutrients. (2020) 12:3118. doi: 10.3390/nu12103118, 33065985 PMC7599709

[3] Ordovas JM Ferguson LR Tai ES Mathers JC. Personalised nutrition and health. BMJ. (2018) 361:bmj.k2173. doi: 10.1136/bmj.k2173, 29898881 PMC6081996

[4] Ramos-Lopez O Assmann TS Astudillo Muñoz EY Baquerizo-Sedano L Barrón-Cabrera E Bernal CA . Guidance and position of RINN22 regarding precision nutrition and nutriomics. Lifestyle Genom. (2024) 18:1–19. doi: 10.1159/000542789, 39617000 PMC11844698

[5] Camp KM Trujillo E. Position of the academy of nutrition and dietetics: nutritional genomics. J Acad Nutr Diet. (2014) 114:299–312. doi: 10.1016/j.jand.2013.12.001, 24439821

[6] Bush CL Blumberg JB El-Sohemy A Minich DM Ordovás JM Reed DG . Toward the definition of personalized nutrition: a proposal by the American nutrition association. J Am Coll Nutr. (2020) 39:5–15. doi: 10.1080/07315724.2019.1685332 31855126

[7] Nehlig A. Interindividual differences in caffeine metabolism and factors driving caffeine consumption. Pharmacol Rev. (2018) 70:384–411. doi: 10.1124/pr.117.014407 29514871

[8] De Caterina R El-Sohemy A. Moving towards specific nutrigenetic recommendation algorithms: caffeine, genetic variation and cardiovascular risk. Lifestyle Genom. (2016) 9:106–15. doi: 10.1159/000446801 27467525

[9] Lemaitre RN Tanaka T Tang W Manichaikul A Foy M Kabagambe EK . Genetic loci associated with plasma phospholipid n-3 fatty acids: a meta-analysis of genome-wide association studies from the CHARGE consortium. PLoS Genet. (2011) 7:e1002193. doi: 10.1371/journal.pgen.1002193, 21829377 PMC3145614

[10] Chilton F Murphy R Wilson B Sergeant S Ainsworth H Seeds M . Diet-gene interactions and PUFA metabolism: a potential contributor to health disparities and human diseases. Nutrients. (2014) 6:1993–2022. doi: 10.3390/nu6051993, 24853887 PMC4042578

[11] Roke K Walton K Klingel S Harnett A Subedi S Haines J . Evaluating changes in omega-3 fatty acid intake after receiving personal FADS1 genetic information: a randomized nutrigenetic intervention. Nutrients. (2017) 9:240. doi: 10.3390/nu9030240, 28272299 PMC5372903

[12] Sokary S Almaghrbi H Bawadi H. The interaction between body mass index genetic risk score and dietary intake on weight status: a systematic review. DMSO. (2024) 17:925–41. doi: 10.2147/DMSO.S452660, 38435632 PMC10908334

[13] Jang M-J Tan L-J Park MY Shin S Kim J-M. Identification of interactions between genetic risk scores and dietary patterns for personalized prevention of kidney dysfunction in a population-based cohort. Nutr Diabetes. (2024) 14:62. doi: 10.1038/s41387-024-00316-z, 39143076 PMC11325018

[14] Brennan L De Roos B. Role of metabolomics in the delivery of precision nutrition. Redox Biol. (2023) 65:102808. doi: 10.1016/j.redox.2023.102808, 37423161 PMC10461186

[15] Trouwborst I Gijbels A Jardon KM Siebelink E Hul GB Wanders L . Cardiometabolic health improvements upon dietary intervention are driven by tissue-specific insulin resistance phenotype: a precision nutrition trial. Cell Metab. (2023) 35:e5:71–83. doi: 10.1016/j.cmet.2022.12.002, 36599304

[16] De Vienne D. What is a phenotype? History and new developments of the concept. Genetica. (2022) 150:153–8. doi: 10.1007/s10709-021-00134-6, 34739647

[17] Hillesheim E Yin X Sundaramoorthy GP Brennan L. Using a metabotype framework to deliver personalized nutrition improves dietary quality and metabolic health parameters: a 12-week randomized controlled trial. Mol Nutr Food Res. (2023) 67:e2200620. doi: 10.1002/mnfr.202200620, 37038841

[18] Hughes RL Kable ME Marco M Keim NL. The role of the gut microbiome in predicting response to diet and the development of precision nutrition models. Part II: results. Adv Nutr. (2019) 10:979–98. doi: 10.1093/advances/nmz049, 31225587 PMC6855959

[19] Pires L Gonzalez-Paramás AM Heleno SA Calhelha RC. Gut microbiota as an endocrine organ: unveiling its role in human physiology and health. Appl Sci. (2024) 14:9383. doi: 10.3390/app14209383

[20] Briazu RA Bell L Dodd GF Blackburn S Massri C Chang B . The effectiveness of personalised food choice advice tailored to an individual’s socio-demographic, cognitive characteristics, and sensory preferences. Appetite. (2024) 201:107600. doi: 10.1016/j.appet.2024.107600, 39002566

[21] Renner B Buyken AE Gedrich K Lorkowski S Watzl B Linseisen J . Perspective: a conceptual framework for adaptive personalized nutrition advice systems (APNASs). Adv Nutr. (2023) 14:983–94. doi: 10.1016/j.advnut.2023.06.009, 37419418 PMC10509404

[22] Dai X Shen L. Advances and trends in omics technology development. Front Med. (2022) 9:911861. doi: 10.3389/fmed.2022.911861, 35860739 PMC9289742

[23] Chaudhary N Kumar V Sangwan P Pant NC Saxena A Joshi S . "Personalized nutrition and -omics". In: Comprehensive Foodomics (Elsevier) (2021). p. 495–507. doi: 10.1016/B978-0-08-100596-5.22880-1

[24] Shinn LM Holscher HD. Personalized nutrition and multiomics analyses: a guide for nutritionists. Nutr Today. (2021) 56:270–8. doi: 10.1097/NT.0000000000000513

[25] Donovan SM Abrahams M Anthony JC Bao Y Barragan M Bermingham KM . Personalized nutrition: perspectives on challenges, opportunities, and guiding principles for data use and fusion. Crit Rev Food Sci Nutr. (2025) 65:7151–69. doi: 10.1080/10408398.2025.2461237, 39907017

[26] Eichelmann F Prada M Sellem L Jackson KG Salas Salvadó J Razquin Burillo C . Lipidome changes due to improved dietary fat quality inform cardiometabolic risk reduction and precision nutrition. Nat Med. (2024) 30:2867–77. doi: 10.1038/s41591-024-03124-1, 38992128 PMC11485259

[27] Zeevi D Korem T Zmora N Israeli D Rothschild D Weinberger A . Personalized nutrition by prediction of glycemic responses. Cell. (2015) 163:1079–94. doi: 10.1016/j.cell.2015.11.001, 26590418

[28] Al-Amrani S Al-Jabri Z Al-Zaabi A Alshekaili J Al-Khabori M. Proteomics: concepts and applications in human medicine. WJBC. (2021) 12:57–69. doi: 10.4331/wjbc.v12.i5.57, 34630910 PMC8473418

[29] Graves PR Haystead TAJ. Molecular biologist’s guide to proteomics. Microbiol Mol Biol Rev. (2002) 66:39–63. doi: 10.1128/MMBR.66.1.39-63.2002, 11875127 PMC120780

[30] Kussmann M Panchaud A Affolter M. Proteomics in nutrition: status quo and outlook for biomarkers and bioactives. J Proteome Res. (2010) 9:4876–87. doi: 10.1021/pr1004339, 20718507

[31] Cui M Cheng C Zhang L. High-throughput proteomics: a methodological mini-review. Lab Investig. (2022) 102:1170–81. doi: 10.1038/s41374-022-00830-7, 35922478 PMC9362039

[32] Aslam B Basit M Nisar MA Khurshid M Rasool MH. Proteomics: technologies and their applications. J Chromatogr Sci. (2017) 55:182–96. doi: 10.1093/chromsci/bmw167, 28087761

[33] Wasinger VC Zeng M Yau Y. Current status and advances in quantitative proteomic mass spectrometry. Int J Proteom. (2013) 2013:1–12. doi: 10.1155/2013/180605, 23533757 PMC3606794

[34] Aparna GM Tetala KKR. Recent Progress in development and application of DNA, protein, peptide, glycan, antibody, and Aptamer microarrays. Biomolecules. (2023) 13:602. doi: 10.3390/biom13040602, 37189350 PMC10135839

[35] Liang J Tian J Zhang H Li H Chen L. Proteomics: an in-depth review on recent technical advances and their applications in biomedicine. Med Res Rev. (2025) 45:1021–44. doi: 10.1002/med.22098, 39789883

[36] Wang S Chu Y Yuan J Li Y Liu Z Chen X . Application and prospects of proteomic technology in inflammation: a review. Food Sci Human Wellness. (2024) 13:2373–85. doi: 10.26599/FSHW.2022.9250248

[37] Kussmann M. Protein matters and proteins matter: proteomics and peptidomics in nutrition and health. Nutrients. (2023) 15:2762. doi: 10.3390/nu15122762, 37375666 PMC10303989

[38] Wang J Li D Dangott LJ Wu G. Proteomics and its role in nutrition research. J Nutr. (2006) 136:1759–62. doi: 10.1093/jn/136.7.1759, 16772433

[39] Sauer S Luge T. Nutriproteomics: facts, concepts, and perspectives. Proteomics. (2015) 15:997–1013. doi: 10.1002/pmic.201400383, 25475255

[40] Ganesh V Hettiarachchy NS. Nutriproteomics: a promising tool to link diet and diseases in nutritional research. Biochim Biophys Acta (BBA) Prot Proteom. (2012) 1824:1107–17. doi: 10.1016/j.bbapap.2012.06.006, 22732351

[41] Rodriguez-Muñoz A Motahari-Rad H Martin-Chaves L Benitez-Porres J Rodriguez-Capitan J Gonzalez-Jimenez A . A systematic review of proteomics in obesity: unpacking the molecular puzzle. Curr Obes Rep. (2024) 13:403–38. doi: 10.1007/s13679-024-00561-4, 38703299 PMC11306592

[42] Page MJ McKenzie JE Bossuyt PM Boutron I Hoffmann TC Mulrow CD . The PRISMA 2020 statement: an updated guideline for reporting systematic reviews. BMJ. (2021). 372:n71. doi: 10.1136/bmj.n71, 33782057 PMC8005924

[43] Amir-Behghadami M Janati A. Population, intervention, comparison, outcomes and study (PICOS) design as a framework to formulate eligibility criteria in systematic reviews. Emerg Med J. (2020) 37:387. doi: 10.1136/emermed-2020-209567, 32253195

[44] Sterne JAC Savović J Page MJ Elbers RG Blencowe NS Boutron I . RoB 2: a revised tool for assessing risk of bias in randomised trials. BMJ. (2019):l4898. doi: 10.1136/bmj.l4898, 31462531

[45] Grytten E Laupsa-Borge J Cetin K Bohov P Nordrehaug JE Skorve J . Inflammatory markers after supplementation with marine n-3 or plant n-6 PUFAs: a randomized double-blind crossover study. J Lipid Res. (2025) 66:100770. doi: 10.1016/j.jlr.2025.100770, 40058591 PMC11999210

[46] Sim M Hong S Jung MH Choi EY Hwang G-S Shin D-M . Gut microbiota links vitamin C supplementation to enhanced mental vitality in healthy young adults with suboptimal vitamin C status: a randomized, double-blind, placebo-controlled trial. Brain Behav Immun. (2025) 128:179–91. doi: 10.1016/j.bbi.2025.03.032, 40187667

[47] Saadati S De Courten M Deceneux C Plebanski M Scott D Mesinovic J . Carnosine supplementation has no effect on inflammatory markers in adults with prediabetes and type 2 diabetes: a randomised controlled trial. Nutrients. (2024) 16:3900. doi: 10.3390/nu16223900, 39599686 PMC11597812

[48] Erta G Gersone G Jurka A Tretjakovs P. Impact of a 12-week dietary intervention on adipose tissue metabolic markers in overweight women of reproductive age. IJMS. (2024) 25:8512. doi: 10.3390/ijms25158512, 39126081 PMC11313195

[49] Simpson AMR De Souza MJ Damani J Rogers CJ Williams NI Weaver CM . Gut microbes differ in postmenopausal women responding to prunes to maintain hip bone mineral density. Front Nutr. (2024) 11:1389638. doi: 10.3389/fnut.2024.1389638, 38706560 PMC11067506

[50] Hande LN Thunhaug H Ludviksen J Hovland A Lappegård KT. No effect of omega-3 polyunsaturated fatty acid supplementation on inflammatory markers in familial hypercholesterolemia: a randomized crossover trial. Scand J Clin Lab Invest. (2023) 83:152–9. doi: 10.1080/00365513.2023.2178499, 36999528

[51] Ying Q Croyal M Chan DC Blanchard V Pang J Krempf M . Effect of omega-3 fatty acid supplementation on the postprandial metabolism of Apolipoprotein(a) in familial hypercholesterolemia. JAT. (2023) 30:274–86. doi: 10.5551/jat.63587, 35676030 PMC9981347

[52] Lancaster SM Lee-McMullen B Abbott CW Quijada JV Hornburg D Park H . Global, distinctive, and personal changes in molecular and microbial profiles by specific fibers in humans. Cell Host Microbe. (2022) 30:848–862.e7. doi: 10.1016/j.chom.2022.03.036, 35483363 PMC9187607

[53] Hatairaktham S Masaratana P Hantaweepant C Srisawat C Sirivatanauksorn V Siritanaratkul N . Curcuminoids supplementation ameliorates iron overload, oxidative stress, hypercoagulability, and inflammation in non-transfusion-dependent β-thalassemia/Hb E patients. Ann Hematol. (2021) 100:891–901. doi: 10.1007/s00277-020-04379-7, 33388858

[54] Yusin J Wang V Henning SM Yang J Tseng C-H Thames G . The effect of broccoli sprout extract on seasonal grass pollen-induced allergic rhinitis. Nutrients. (2021) 13:1337. doi: 10.3390/nu13041337, 33920642 PMC8074067

[55] Kanoni S Kumar S Amerikanou C Kurth MJ Stathopoulou MG Bourgeois S . Nutrigenetic interactions might modulate the antioxidant and anti-inflammatory status in Mastiha-supplemented patients with NAFLD. Front Immunol. (2021) 12:683028. doi: 10.3389/fimmu.2021.683028, 34025683 PMC8138178

[56] Yubero-Serrano EM Fernandez-Gandara C Garcia-Rios A Rangel-Zuñiga OA Gutierrez-Mariscal FM Torres-Peña JD . Mediterranean diet and endothelial function in patients with coronary heart disease: an analysis of the CORDIOPREV randomized controlled trial. PLoS Med. (2020) 17:e1003282. doi: 10.1371/journal.pmed.1003282, 32903262 PMC7480872

[57] Macnaughtan J Figorilli F García-López E Lu H Jones H Sawhney R . A double-blind, randomized placebo-controlled trial of probiotic *Lactobacillus casei* Shirota in stable cirrhotic patients. Nutrients. (2020) 12:1651. doi: 10.3390/nu12061651, 32498372 PMC7352321

[58] Paganini D Uyoga MA Kortman GAM Boekhorst J Schneeberger S Karanja S . Maternal human milk oligosaccharide profile modulates the impact of an intervention with iron and galacto-oligosaccharides in Kenyan infants. Nutrients. (2019) 11:2596. doi: 10.3390/nu11112596, 31671757 PMC6893608

[59] Rodríguez-Cruz M Cruz-Guzmán ODR Almeida-Becerril T Solís-Serna AD Atilano-Miguel S Sánchez-González JR . Potential therapeutic impact of omega-3 long chain-polyunsaturated fatty acids on inflammation markers in Duchenne muscular dystrophy: a double-blind, controlled randomized trial. Clin Nutr. (2018) 37:1840–51. doi: 10.1016/j.clnu.2017.09.011, 28987470

[60] Korpela K Salonen A Vepsäläinen O Suomalainen M Kolmeder C Varjosalo M . Probiotic supplementation restores normal microbiota composition and function in antibiotic-treated and in caesarean-born infants. Microbiome. (2018) 6:182. doi: 10.1186/s40168-018-0567-4, 30326954 PMC6192119

[61] Ali A Weiss TR McKee D Scherban A Khan S Fields MR . Efficacy of individualised diets in patients with irritable bowel syndrome: a randomised controlled trial. BMJ Open Gastroenterol. (2017) 4:e000164. doi: 10.1136/bmjgast-2017-000164, 29018540 PMC5628288

[62] Waldvogel S Rochat B Peduzzi D Vaucher P Tissot J-D Favrat B. Effects of oral supplementation of iron on hepcidin blood concentrations among non-anaemic female blood donors: a randomized controlled trial. Vox Sang. (2016) 110:166–71. doi: 10.1111/vox.12348, 26394360

[63] Chase HP Boulware D Rodriguez H Donaldson D Chritton S Rafkin-Mervis L . The type 1 diabetes TrialNet nutritional intervention to prevent (NIP) type 1 diabetes study group. Effect of docosahexaenoic acid supplementation on inflammatory cytokine levels in infants at high genetic risk for type 1 diabetes: DHA and infant inflammatory cytokines. Pediatr Diabetes. (2015) 16:271–9. doi: 10.1111/pedi.12170, 25039804 PMC4291300

[64] De Luis DA Izaola O De La Fuente B Primo D Romero E. Role of fatty acid-binding protein 2 Ala54Thr genotype on weight loss and cardiovascular risk factors after a high-protein/low-carbohydrate versus a standard hypocaloric diet during 9 months. Ann Nutr Metab. (2015) 67:81–6. doi: 10.1159/000438947, 26316042

[65] Heng EC Karsani SA Abdul Rahman M Abdul Hamid NA Hamid Z Wan Ngah WZ. Supplementation with tocotrienol-rich fraction alters the plasma levels of Apolipoprotein A-I precursor, Apolipoprotein E precursor, and C-reactive protein precursor from young and old individuals. Eur J Nutr. (2013) 52:1811–20. doi: 10.1007/s00394-012-0485-3, 23287846

[66] Barona J Blesso C Andersen C Park Y Lee J Fernandez M. Grape consumption increases anti-inflammatory markers and upregulates peripheral nitric oxide synthase in the absence of dyslipidemias in men with metabolic syndrome. Nutrients. (2012) 4:1945–57. doi: 10.3390/nu4121945, 23222963 PMC3546615

[67] Rubio-Aliaga I Marvin-Guy LF Wang P Wagniere S Mansourian R Fuerholz A . Mechanisms of weight maintenance under high- and low-protein, low-glycaemic index diets. Mol Nutr Food Res. (2011) 55:1603–12. doi: 10.1002/mnfr.201100081, 21957032

[68] Brauer HA Libby TE Mitchell BL Li L Chen C Randolph TW . Cruciferous vegetable supplementation in a controlled diet study alters the serum peptidome in a GSTM1-genotype dependent manner. Nutr J. (2011) 10:11. doi: 10.1186/1475-2891-10-11, 21272319 PMC3042379

[69] Lehtonen H-M Suomela J-P Tahvonen R Yang B Venojärvi M Viikari J . Different berries and berry fractions have various but slightly positive effects on the associated variables of metabolic diseases on overweight and obese women. Eur J Clin Nutr. (2011) 65:394–401. doi: 10.1038/ejcn.2010.268, 21224867

[70] Kim JY Paik JK Kim OY Park HW Lee JH Jang Y . Effects of lycopene supplementation on oxidative stress and markers of endothelial function in healthy men. Atherosclerosis. (2011) 215:189–95. doi: 10.1016/j.atherosclerosis.2010.11.036, 21194693

[71] Jiang Y Wang J Sun A Zhang H Yu X Qin W . The coming era of proteomics-driven precision medicine. Natl Sci Rev. (2025) 12:nwaf278. doi: 10.1093/nsr/nwaf278, 40842868 PMC12365760

[72] Araújo R Von Rekowski CP Fonseca TAH Calado CRC Ramalhete L Bento L. Multiplex targeted proteomic analysis of cytokine ratios for ICU mortality in severe COVID-19. Proteomes. (2025) 13:35. doi: 10.3390/proteomes13030035, 40843708 PMC12372026

[73] Numis AL Fox CH Lowenstein DJ Norris PJ Di Germanio C. Comparison of multiplex cytokine assays in a pediatric cohort with epilepsy. Heliyon. (2021) 7:e06445. doi: 10.1016/j.heliyon.2021.e06445, 33748497 PMC7966851

[74] Sonbol HS. Nutritional proteomics: a key to unlocking optimal human health. Arch Pharm Pract. (2024) 15:68–83. doi: 10.51847/nko14dBXgB

[75] Okawa T Nagai M Hase K. Dietary intervention impacts immune cell functions and dynamics by inducing metabolic rewiring. Front Immunol. (2021) 11:623989. doi: 10.3389/fimmu.2020.623989, 33613560 PMC7890027

[76] Zhang J Dai W Chen Y. Editorial: the roles of lipids in immunometabolism: the crosstalk between lipid metabolisms and inflammation. Front Cardiovasc Med. (2022) 9:938535. doi: 10.3389/fcvm.2022.938535, 35811702 PMC9257247

[77] Sobsey CA Ibrahim S Richard VR Gaspar V Mitsa G Lacasse V . Targeted and untargeted proteomics approaches in biomarker development. Proteomics. (2020) 20:1900029. doi: 10.1002/pmic.201900029, 31729135

[78] Van Den Bossche T Armengaud J Benndorf D Blakeley-Ruiz JA Brauer M Cheng K . The microbiologist’s guide to metaproteomics. iMeta. (2025) 4:e70031. doi: 10.1002/imt2.70031, 40469504 PMC12130581

[79] Das A Behera RN Kapoor A Ambatipudi K. The potential of meta-proteomics and artificial intelligence to establish the next generation of probiotics for personalized healthcare. J Agric Food Chem. (2023) 71:17528–42. doi: 10.1021/acs.jafc.3c03834, 37955263

[80] Lampe JW Navarro SL Hullar MAJ Shojaie A. Inter-individual differences in response to dietary intervention: integrating omics platforms towards personalised dietary recommendations. Proc Nutr Soc. (2013) 72:207–18. doi: 10.1017/S0029665113000025, 23388096 PMC3694579

[81] Nejadghaderi SA Balibegloo M Rezaei N. The Cochrane risk of bias assessment tool 2 (RoB 2) versus the original RoB: a perspective on the pros and cons. Health Sci Rep. (2024) 7:e2165. doi: 10.1002/hsr2.2165, 38835932 PMC11147813

[82] Verma M Hontecillas R Tubau-Juni N Abedi V Bassaganya-Riera J. Challenges in personalized nutrition and health. Front Nutr. (2018) 5:117. doi: 10.3389/fnut.2018.00117, 30555829 PMC6281760

