# Supplementary material for: Proteomic profiling in personalized nutrition: a systematic review and methodological frameworks of randomized controlled trials
Source: Front Nutr. 2026 Jun 10;13:1826381. doi: 10.3389/fnut.2026.1826381 (PMC13290611; doi:10.3389/fnut.2026.1826381)
Supplement: Supplementary file 1 [file Table_1.DOCX]

Supplementary Material

# Research strategy

In PubMed and Embase, the following search string was used:

("proteomic*" OR "proteome" OR "protein analysis" OR "protein isolation" OR "circulating protein*" OR "plasma* protein*" OR "protein profile*" OR "protein isoform*" OR "protein level*" OR "protein expression" OR "protein change*" OR "protein modification*" OR "post-translation* modification*" OR "protein function*" OR "protein interaction*" OR "protein activit*" OR "enzym* level*" OR "enzym* analysis" OR "enzym* profile*" OR "enzym* activit*" OR "cytokine* level*" OR "cytokine* profile*" OR "peptid* analysis" OR "peptid* profil*" OR "peptid* level*" OR "peptid* modification*" OR "plasma* peptide*" OR "protein array*" OR "proteomic chip" OR "protein assay*" OR "mass spectrometry" OR "liquid chromatography" OR "LC-MS/MS" OR "bead based" OR "aptamer based" OR "multiplex immunoassay*" OR "SOMAscan" OR "Olink") AND ("tailored nutrition*" OR "precision diet*" OR "precision nutrition*" OR "personalized diet*" OR "personalized nutrition*" or "personalized intervention*" OR "tailored intervention*" OR "nutrigenomic*" OR "individualized nutrition*" OR "individualized diet*" OR "medical nutrition therap*" OR "diet* therapy" OR "diet* counsel*" OR "nutrition* counsel*" OR "counselling" OR "genotyp*" OR "metabotyp*" OR "phenotyp*" OR "diet* supplement*") AND ("diet*" OR "nutrition*").

In Scopus, the following search string was used:

TITLE-ABS-KEY ( ( "proteomic*" OR "proteome" OR "protein analysis" OR "protein isolation" OR "circulating protein*" OR "plasma* protein*" OR "protein profile*" OR "protein isoform*" OR "protein level*" OR "protein expression" OR "protein change*" OR "protein modification*" OR "post-translation* modification*" OR "protein function*" OR "protein interaction*" OR "protein activit*" OR "enzym* level*" OR "enzym* analysis" OR "enzym* profile*" OR "enzym* activit*" OR "cytokine* level*" OR "cytokine* profile*" OR "peptid* analysis" OR "peptid* profil*" OR "peptid* level*" OR "peptid* modification*" OR "plasma* peptide*" OR "protein array*" OR "proteomic chip" OR "protein assay*" OR "mass spectrometry" OR "liquid chromatography" OR "LC-MS/MS" OR "bead based" OR "aptamer based" OR "multiplex immunoassay*" OR "SOMAscan" OR "Olink" ) AND ( "tailored nutrition*" OR "precision diet*" OR "precision nutrition*" OR "personalized diet*" OR "personalized nutrition*" OR "personalized intervention*" OR "tailored intervention*" OR "nutrigenomic*" OR "individualized nutrition*" OR "individualized diet*" OR "medical nutrition therap*" OR "diet* therapy" OR "diet* counsel*" OR "nutrition* counsel*" OR "genotyp*" OR "metabotyp*" OR "phenotyp*" OR "diet* supplement*" ) AND ( "diet*" OR "nutrition*" ) AND ( "randomized controlled trial" OR "RCT" OR "randomised controlled trial" ) ) AND ( LIMIT-TO ( SRCTYPE , "j" ) ) AND ( LIMIT-TO ( DOCTYPE , "ar" ) ). Filters were applied to limit results to studies published between 2010 and 2025, producing 2,409 results.

In all databases, filters were applied to limit results to studies published between 2010 and 2025 and to randomized controlled trials.

# Supplementary Figures


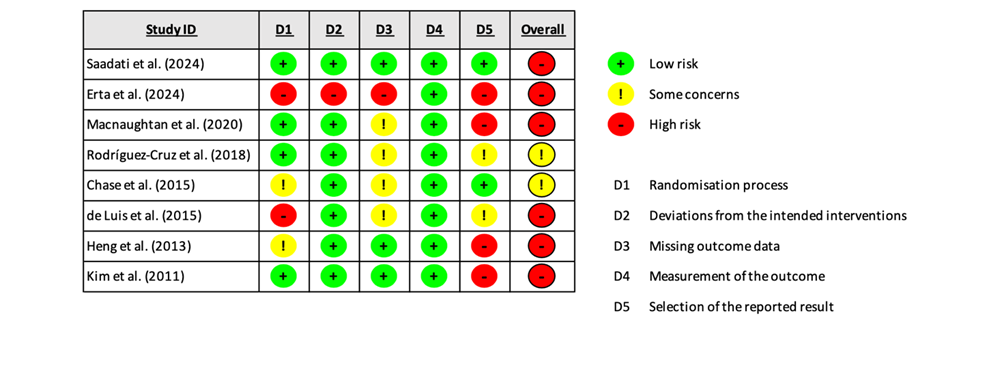
**Supplementary Figure 1.** Detailed study-by-study Risk of Bias summary in parallel RCTs (Intention-to-treat).


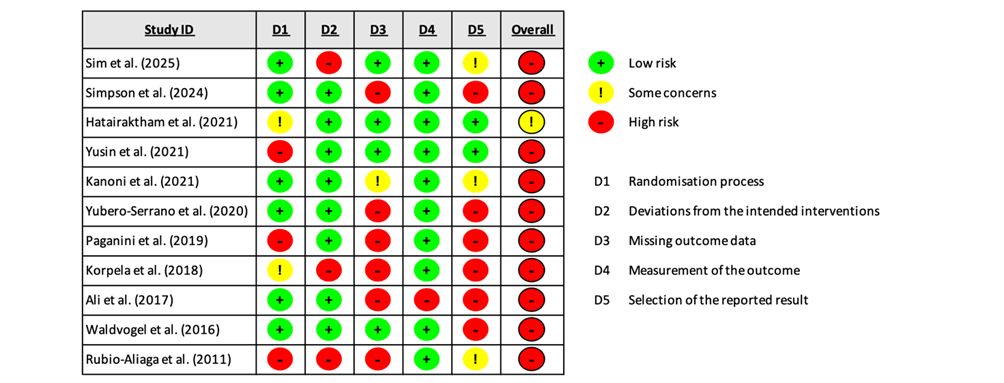


**Supplementary Figure 2.** Detailed study-by-study Risk of Bias summary in parallel RCTs (Per-protocol).
