# Supplementary material for: Proteomic profiling in personalized nutrition: a systematic review and methodological frameworks of randomized controlled trials
Source: Front Nutr. 2026 Jun 10;13:1826381. doi: 10.3389/fnut.2026.1826381 (PMC13290611; doi:10.3389/fnut.2026.1826381)
Supplement: Supplementary file 2 [file Table_2.docx]

| **Ref** | **Study Design [study settings; study type]** | **General characteristics** | **Intervention design** | **Control** | **Personalization approach** | **Proteomics [Targeted/ Untargeted]** | **Biomarkers [biological sample]** | **Main findings** |
| --- | --- | --- | --- | --- | --- | --- | --- | --- |
| Grytten et al. (45) | Randomized, controlled, double blind, crossover, two 7-week intervention periods separated by a 9-week washout phase. [single-centre; secondary analysis] | Healthy subjects with increased waistline and an overall sedentary lifestyle. (n = 39)  Sex: F=16, M=23 Age: 56±9.3 y BMI: F = 28.5±4.5 kg/m2 M = 29.8±3.8 kg/m^2^ | **Supplementation-based:  ω-3 PUFA**   ~~(~~3–4 g/d EPA/DHA from fish oil for 7 weeks) | **Supplementation-based:**  **ω-6 PUFA**   ~~(~~15–20 g/d LA from safflower oil for 7 weeks)  ~~.~~ | **Phenotype-based stratification: exploratory analysis**  · Stratification based on **ω-3 index**:  - **Low**: <8 wt% (n = 14) - **High**: ≥8 wt% (n = 24);   · Stratification based on **LA index**:  - **Low**: < (8.88 wt%) (n = 18) - **High**: > 8.88 wt% (n = 20) | Bio-Plex Pro Human Cytokine Assay (Bio-Rad)  [Targeted];  MALDI-TOF mass spectrometry at Bevital AS, Norway. [Targeted] | Cytokines, chemokines, including eotaxin, MCP-1, MIP-1α, MIP-1β, RANTES, TNF-α, IL-1RA, IL-8 [serum]; acute-phase proteins: SAA (SAAt, SAA1t, SAA2t), calprotectin (S100t, S100A8, S100A9), hs-CRP [plasma]. | After ω-6 supplementation · Stratifying by **ω-3 index** - ↓Eotaxin (p = 0.030), ↓MCP-1 (p = 0.050) in high vs low n-3 index; - no significant between-group differences;  - within-treatment (high n-3 index): ↓MCP-1, MIP-1β, RANTES, TNF ;↑ CRP, SAAt, SAA1t, SAA2t. · Stratifying by **LA index** - ↓TNF (p = 0.026) in low vs high LA index; - within-group (low LA): ↓MCP-1, RANTES, IL-1RA. After ω-3 supplementation · Stratifying by **ω-3 index** - no significant between low and high n-3 index; · Stratifying by **LA index** - within-treatment (low LA): ↓RANTES  - within-treatment (low and high LA):↓MIP-1β and TNF. After adjustment: only MCP-1 showed a within-treatment ↓ after ω-3 (p = 0.043). |
| Sim et al. (46) | Randomized controlled, double blind, 2-arm parallel, 4-week intervention [single-centre; secondary analysis] | Healthy individuals aged 20–39 years with suboptimal vitamin C levels at baseline. (n = 40)  Vitamin C group (n = 21) Sex: F = 8, M = 13  Age: 25.0±3.5 y  BMI: 22.7±3.1 kg/m^2^  Placebo group (n = 19) Sex: F = 8, M = 11  Age: 23.8±1.9y BMI: 22.6±3.4 kg/m^2^ | **Supplementation-based: Vitamin C**   (two pouches daily of 100 ml vitamin C drink for 4 weeks). | **Placebo administration**  (two pouches daily of 100 ml placebo drink for 4 weeks). | **Phenotype-based stratification: exploratory analysis**  Vitamin C group stratification based on the **changes in *Desulfovibrio* abundance**: - **Non-responders**: <1.5-fold decrease (n = 8); - **Responders**: > 1.5-fold decrease (n = 13). | V-PLEX Proinflammatory Panel 1 Human Kit (Mesoscale Discovery, Rockville, MD, USA).  [Targeted] | IFN-γ, IL-1β, IL-2, IL-4, IL-6, IL-8, IL-10, IL-12p70, IL-13, TNF-⍺ [serum]. | In most samples, IL-1β, IL-2, IL-4, IL-12p70, and IL-13 were below the lower limit of quantitation; these cytokines were excluded from analyses.  ↓IL-10 (p=0.04) in **responder**.  Non-significant:  - IL-6 decrease in responder vs increase in non-responder (p=0.083)  - IL-8 decrease in both groups - IFN-γ decrease in responder vs increase in non- responder (p = 0.215) - TNF-α decrease in both groups (p = 0.401). After correlation analysis: significant positive associations between changes in *Desulfovibrio* abundance and IL-10 concentrations (rs = 0.379, p = 0.015). |
| Saadati et al. (47) | Randomized controlled, quadruple blind, 2-arm parallel, 14-week intervention [single-centre; secondary analysis] | Adults with prediabetes and well-controlled type 2 diabetes (DMT2), either treated with diet or metformin only. (n = 41)  Carnosine group (n = 19) Sex: F = 6, M = 13  Age: 54.5 (45.4, 59.5) y  BMI: 29.9±4.9 kg/m^2^ Prediabetic: n = 11 Diabetic: n = 8 Placebo group (n = 22) Sex: F = 6, M = 16  Age: 50.2 (42.1, 59.3) y BMI: 28.9±3.1 kg/m^2^ Prediabetic: n = 11 Diabetic: n = 11  *Age reported as [median (IQR), years]* | **Supplementation-based: Carnosine**  (two capsules of 500 mg carnosine twice daily for 14 weeks). | **Placebo administration**  (two methylcellulose placebo capsules twice daily for 14 weeks). | **Phenotype-based stratification: baseline stratification**  Stratification based on **glycaemic** **status**: · **Prediabetes** - carnosine (n = 11) - placebo (n = 11) · **Diabetes** - carnosine (n = 8) - placebo (n = 11);  Stratification based on **therapy**:  · **Metformin** - carnosine (n = 7) - placebo (n = 9) · **diet only** - carnosine (n = 12) - placebo (n = 13). | Multiplex assays (Bioplex 200 array reader, Bio-Rad Laboratories, Hercules, CA, USA) - 10-plex obesity panel (catalog # LOBM000, R&D systems, Minneapolis, MN, USA).  [Targeted] | Adiponectin, adipsin, resistin, PAI-1, leptin, CRP, IL-6, IL-10, MCP-1, TNF-α. [serum]. | No significant findings. |
| Erta et al. (48) | Randomized, controlled, 3-arm parallel, 12-week intervention [single-centre; original study] | Healthy women aged 18–45 years with a BMI between 25 and 29.9 kg/m^2^. (n = 76)  Age: 29.5±6.2 y  BMI: 27.8±2.1 kg/m^2^ | **Dietary intervention:**  **Low-starch diet** (LS,  for 12 weeks). | **Dietary intervention:**  **Caloric Restriction diet** (CR, for 12 weeks.) | **Phenotype-based stratification: baseline stratification**  Stratification based on baseline **salivary amylase activity**: · **high-salivary-amylase** (HSA) - HSA-CR (n = 15) - HSA-LS (n = 15); · **low-salivary-amylase** (LSA) - LSA-CR (n = 15) - LSA-LS (n = 15). | Multiplex immunoassay (Luminex, Austin, TX, USA)  [Targeted] | Leptin [plasma]. | · CR group - **HSA**: ↓from T0 to T2 [T0: 7146 pg/mL (4879–13,123); T2: 5607 pg/mL (4452–10,536)] * ; - **LSA**: ↓from T0 to T2. · LS group - **HSA** no significant change; - **LSA** no significant change.  At T1 - Both HSA: increase; - Both LSA: more pronounced increase.  **[Median (IQR)]* |
| Simpson et al. (49) | Randomized, controlled, outcome-assessor– and analyst-blinded,  3-arm parallel,  12-month intervention  [single centre; secondary analysis] | Healthy postmenopausal women aged 55–75 yearswith bone mineral density (BMD) T-scores between 0.0 and −3.0.  (n = 52)  Responders (n = 20)  Age: 62.4±5.6 y  BMI: 24.9±3.7 kg/m^2^  Non-Responders (n = 32)  Age: 62.5±4.6 y  BMI: 26.6±4.4 kg/m^2^ | **Food-based intervention: Prune** (**50 g/day** or **100 g/day** for 12 months)**.** | **Control group** (No prune consumption for 12 months). | **Phenotype-based stratification: exploratory analysis**  Stratification, based on **BMD response at the total hip**: - **Responders**: ≥ +1.0% increase in BMD from baseline (n = 20): - 50g group (n = 13)  - 100g group (n = 7);  - **Non-responders**: ≥ −1.0% decrease in BMD (n = 32): - 50g group (n = 20) - 100g group (n = 12). | V-PLEX Proinflammatory Panel 1 Human Kit and V-PLEX Human MCP-1 kit (Meso Scale Diagnostics, LLC, Rockville, MD).  [Targeted] | IL-1β, IL-6, IL-8, TNF-α, MCP-1  [Plasma; supernatants harvested from lipopolysaccharide-stimulated (LPS) peripheral blood mononuclear cells (PBMCs)]. | ↓ IL-1β in **responders** vs non-responders (p=0.002)  ↓ TNF-α in **responders** vs non-responders (p=0.027)  No other inflammatory markers differed significantly between groups**.** |
| Hande (50) | Randomized, controlled, double blind, cross-over, two treatment periods of 3 months separated by a washout period of 3 months, intervention [single-centre; secondary analysis] | Adults with mutation-positive heterozygous familial hypercholesterolemia (FH) and a statin treatment >12 months.  (n = 34)  Sex: F = 17, M = 17 Age: 46.6 (18-71) y BMI: 27.6 ± 5.0 kg/m^2^  *Age reported as [median (range), years]* | **Supplementation-based:  ω-3 PUFA**  (two capsules twice daily (1840 mg/d eicosapentaenoic acid and 1520 mg/d docosahexaenoic acid); for 3months). | **Placebo administration**   (two olive oil capsules daily for 3 months). | **Genotype-based stratification: inclusion criterion**   Total population composed by individuals with **mutation-positive heterozygous familial hypercholesterolemia (FH).** | Bio-Plex Human Cytokine Grp I Panel 27-Plex (Bio-Rad Laboratories Inc, Hercules, CA). [Targeted] | IL-β, IL-1ra, IL-2, IL-4, IL-5, IL-6, IL-7, IL-8, IL-9, IL-10, IL-12, IL-13, IL-15, IL-17, eotaxin-1, FGF basic, G-CSF, GM- CSF, IFN-γ, CXCL10, MCP-1, MIP-1𝛼, MIP-1β, PDGF-BB, RANTES, TNF, VEGF [Plasma]. | 21 cytokines excluded due to values below the lower detection limit or out of range.   No significant treatment effects observed on the remaining cytokines after correction for multiple comparisons; IL-1β difference (p = 0.04) was not significant after adjustment. |
| Ying et al. (51) | Randomized, controlled, open-label, cross-over, two 8-week interventions separated by a 8-week washout phase [single-centre; secondary analysis] | Adults with mutation-positive heterozygous FH or FH defined by a Dutch Lipid Clinic Network criteria score of more than 8, on statins (or statin + ezetimibe). (n = 20)  Sex: F = 10, M = 10 Age: 53.3±3.0 y  BMI: 27.0±1.4 kg/m2 FH diagnosed by: - Genotype: n=17 - Phenotye: n=3 | **Supplementation-based:  ω-3 PUFA**   (4 g/d for 8 weeks). | **Standard care**    (no ω-3 supplementation for 8 weeks). | **Genotype-based stratification: inclusion criterion**    Population composed by individuals with **FH genotype.** **(3 out of 20 were categorized based on phenotype)* | LC-MS/MS. [Targeted] | Apo(a), apoC-III, apoE (total and LpB fractions) [plasma; plasma triglyceride-rich lipoprotein (TRL) fraction]. | ω-3 supplementation effects: no significant changes in apoE concentration; apoC-III and Lp(a); and in the AUCs of apoC-III and apoE (total and LpB fraction).   Postprandial Responses (ω-3 vs standard care, after a high-fat test meal): - ↓ in total and TRL-apo(a) rise at 0.5 (−17.9%), 1 (−18.7%), 2 (−32.6%), and 3 h (−19.2%) (p<0.05 in all). * - no significant effect on fasting total apo(a) and TRL- apo(a) and postprandial total apo(a) AUC; - ↓ TRL-apo(a) AUC (0–10 h) by 15% (P<0.05).  no significant effect on fasting/postprandial AUC for non-TRL-apo(a) or fasting/postprandial apo(a) in the non-TRL fraction.  ** Data expressed as mean±SEM expressed as relative difference %;* |
| Lancaster et al. (52) | Randomized, controlled, single blind, cross-over,  three 3-week interventions separated by a 6/8-week washout phase [single-centre; original study] | Healthy adults or adults with DMT2 (n = 18)  Sex: F = 10, M = 8 Age: 56.9±7.7 y BMI: - normal: n = 24% - overweight: n = 47%,  - obese: n = 29% Insulin sensitive: n = 62% | **Supplementation-based: Dietary Fibre**    - **Arabinoxylan** (AX, 10 g/d for 3 weeks); - **Long chain Inulin** (LCI, 20g/d for 3 weeks);- **Mixed fibres** (30g/d for 3 weeks). | **Supplementation-based: Dietary Fibre**   - **LCI** (10 g/d for 3 weeks); - **AX** (20g/d for 3 weeks); - **Mixed** **fibres** (30g/d for 3 weeks).  . | **Phenotype-based stratification: exploratory analysis**  Stratification based on **intervention response**: · **Responders**: participants with a significant beneficial response to the supplementation (n = 12):   · **Non-responder**: participants with a minimal or no response to the supplementation (n = 6): | nanoLC-SWATH-MS.  [Untargeted] | No specific protein  [plasma] | After AX supplementation  - Elevated **low-abundance plasma proteins** in **responders**, including cardiac ryanodine receptor (RYR2) and apolipoprotein C (APOC4).  - ↑Liver X receptor (**LXR**) upregulation in **responders** (p = 1.59 × 10⁻³³). - Expression of **FXR-controlled proteins** significantly altered in responders compared to non-responders (p = 2.49 × 10⁻³⁴).  After LCI supplementation and Mixed fibres supplementation: effects not specified. |
| Hatairaktham et al. (53) | Randomized, controlled, 2-arm parallel, 24-week intervention [single-canter; original study] | Non-transfused β0-thalassemia/Hb E (β0βE) patients aged 18–60 years with haemoglobin 5–9 g/dl and serum ferritin >600 ng/ml. (n = 31)  CUR500 group (n = 14) Sex: F = 92.5%, M = 7.5% Age: 36±10 y BMI: 20.9±3.6 kg/m^2^  CUR1000 group (n = 15) Age: 34±11 y BMI: 20.6±4.7 kg/m^2^  Sex: F = 92.5%, M = 7.5% | **Supplementation-based: Curcuminoids**   (500mg/d for 24 weeks). | **Supplementation-based: Curcuminoids**  (1000mg/d for 24 weeks). | **Mixed stratification: inclusion criterion; exploratory analysis** Total population composed by individuals with **non-transfusion-dependent β-thalassemia/Hb E (β⁰-thalassemia/Hb E (β⁰βᴱ) genotype;**  Stratification based on **baseline ferritin**: · ≤ 1000 ng/ml   - CUR500 (n = 7) - CUR1000 (n = 9) · >1000 ng/ml  - CUR500 (n = 7) - CUR1000 (n = 6) | Custom Bio-Plex pro™ human cytokine assay (Bio-Rad Laboratories, Inc., CA). [Targeted] | TNF-α, TGF-β1, IFN-γ, GM-CSF, IL-1β, IL-2, IL-6, IL-8  [Blood, not specified]. | **Effect on genotype-based population:**  both dosages ↓ TNF-α, TGF-β1, IFN-γ, IL-1β, IL-6, and IL-8 (IL-2 and GM-CSF undetectable).  Applying a 20% reduction threshold as a response criterion: the 1000-mg supplementation was associated with higher response rates of inflammatory parameters.  **Effect on baseline phenotype**: cytokines levels were generally reduced in all subgroups.  Applying a 20% reduction threshold as a response criterion: 50% response rate was observed in the 1000 mg/day group with baseline ferritin >1000 ng/ml. |
| Yusin et al. (54) | Randomized, controlled, double blind, 4-arm parallel, 3-week intervention [multi-centre; original study] | Non-smoking US veterans with a history of symptoms consistent with seasonal allergic rhinitis to grass pollen (n = 45)  - **Group 1 (G1)** (n = 16) Sex: F = 25%, M = 75% Age: 45.1±12.2 y - **Group 3 (G3)** (n = 14)* Sex: F = 17%, M = 83% Age: 48.8±15.9 y **total analyzed n=12* - **Group 2 (G2)** (n = 9) Sex: F = 23%, M = 78% Age: 58.6±13.5 y - **Group 4 (G4)** (n = 8) Sex: F = 37%, M = 63% Age: 44.5±18.5 y | **Supplementation-based: Broccoli sprout extract (BSE)**     (four 375mg tablet BSE+ nasal corticosteroid for 3 weeks; four 375mg tablet BSE + saline nasal spray for 3 weeks). | **Placebo administration**    (placebo tablet + nasal corticosteroid for 3 weeks; placebo tablet + saline nasal spray for 3 weeks). | **Genotype-based stratification: baseline stratification**  Stratification based on **Glutathione-S-transferase (GST) polymorphisms:** · **GSTM1**: - **null**: [G1 n = 9, G2 n = 3 G3 n = 4 G4 n = 2]; - **2,3,4**: [G1 n = 5, G2 n = 4, G3 n = 1, G4 n = 2].  · **GSTT1**:  - **null**: [G1 n = 5 G2 n = 4 G3 n = 1 G4 n = 2]; - **1,2**: [G1 n = 11, G2 n = 5, G3 n = 10, G4 n = 6]. · **GSTP1**: - **G/G** [G1 n = 8, G2 n = 4, G3 n = 5, G4 n = 3]; - **A/G** [G1 n = 5, G2 n = 4 G3 n = 4, G4 n = 2]; - **A/A [**G1 n = 3, G2 n = 1, G3 n = 2, G4 n = 3]. | Luminex MagPix® analyzer (Luminex, Austin, TX, USA) - Human cytokine panel HCYTOMAG-60K-06 (EMD Millipore, Billerica, MA, USA).  [Targeted] | IL-1β, IL-4, IL-5, IL-6, IL-8 and IL-13 [Nasal fluid]. | No significant differences in cytokine concentrations among the four groups studied. |
| Kanoni et al. (55) | Randomized, controlled, double blind, 2-arm parallel, 6-month intervention [multi-centre; secondary analysis] | Individuals aged 18-67 years, with BMI≥30 kg/m^2^ and with established NAFLD/NASH.  (n = 98)  - Mastiha group (n = 41) Sex: F = 15, M = 26 Age: 48.66±9.89 y BMI: 34.14±3.38 kg/m^2^ - Placebo group (n = 57) Sex: F = 15, M = 42 Age: 48.95±9.04 y BMI: 34.66±5.05 kg/m^2^ | **Supplementation-based: Mastiha**  (2.1 g/d for 6 months). | **Placebo administration**    (corn starch capsules, 2.1 g/d for 6 months). | **Mixed stratification: exploratory analysis; exploratory analysis**  stratification based on genotype: · IL-6 levels evaluation  - **rs13173271 genotypes** (CC, CT, TT); - **rs4731418 genotypes** (CC, CG, GG). · IL-10 levels evaluation - **rs12173570 genotypes** (CC,CT,TT); - **rs8021058 genotypes** (CC, CT, TT);  Stratification based on **baseline BMI:** · **BMI≤35 kg/m^2^** - **Mastiha** (n = 28) - placebo (n = 37); · **BMI<35 kg/m^2^** - **Mastiha** (n = 13) - placebo (n = 20). | Randox high sensitivity cytokine I multiplex array (Randox Laboratories Ltd, Crumlin, UK). [Targeted] | IL-1α, IL-1β, IL-2, IL-4, IL-6, IL-8, IL-10, MCP-1, TNF-α, INF-γ, EGF, VEGF-A [serum]. | Post-treatment IL6 levels **Carriers of the G allele** for the rs13173271 (n = 18) or the rs4731418 (n = 17), within the Mastiha group, had a trend for lower post-treatment IL-6 levels compared to homozygous of the alternate allele in both groups (n = 33, n = 48).  Post-treatment IL-10 levels: **carriers of the T allele** of the rs12173570 (n = 8) or the **C allele** of the rs8021058 (n =20) within the Mastiha group, had a trend for higher post-treatment IL-10 levels compared to homozygous of the alternate allele in both treatment groups (n = 64, n = 30).  In the **BMI ≤ 35kg/m2 group** (n = 59): increasing trend for the MCP-1 levels within the Mastiha group, non-significant after adjustment for the use of antilipidemic, antihypertensive and/or antidiabetic medication (beta=0.344, SE=0.169, P value=0.047) *.  ** Data expressed as regression coefficients (β ± SE).* |
| Yubero-Serrano et al. (56) | Randomized, controlled, single blind, 2-arm parallel, 7-year intervention [multi-centre; secondary analysis] | Patients aged 20–75 years, with established CHD but without clinical events in the last 6 months, (n= 805)  Mediterranean diet (n = 418) - FMD<2% Sex: F = 16, M = 166 Age: 60.8±0.8 y BMI: 31.1±0.1 kg/m^2^ - FMD≥2% Sex: F = 22, M = 264 Age: 60.0±0.5 y BMI: 30.8±0.28 kg/m^2^ Low-fat diet (n = 387) - FMD<2% Sex: F = 11, M = 110 Age: 60.1±0.8 y BMI: 31.7±0.5 kg/m^2^ - FMD≥2% Sex: F = 12, M = 254 Age: 59.7±0.5 y BMI: 30.8±0.3 kg/m^2^ | **Dietary intervention: Mediterranean diet** (for 1 year). | **Dietary intervention:** **Low-fat and high-complex carbohydrate diet**  (for 1 year). | **Phenotype-based stratification: exploratory analysis**   Stratification based on **baseline endothelial dysfunction**:  - **severe endothelial dysfunction:** FMD < 2%  - Mediterranean diet (n = 132)  - Low low-fat diet (n = 121);  - **non-severe endothelial dysfunction:**  FMD ≥ 2%;  - Mediterranean diet (n = 286)  - Low low-fat diet (n = 266). | nano LC- SWATH-MS.*  [Untargeted]  ** Proteomic analysis applied only on 24 select partecipants* | No specific protein [serum]. | Total proteins identified: n = 224.  **Patients with severe endothelial dysfunction:** - Upregulation of Fibrinogen α chain, haptoglobin-related protein, coagulation factor IX after low-fat diet; - Down-regulation of haemoglobin subunit β, CRP after Mediterranean diet.  **Patients with non-severe endothelial dysfunction: -** Apolipoprotein F and E, glutathione peroxidase 3, and fibrinogen β chain up-regulated and down-regulated after the low-fat diet and the Mediterranean diet, respectively; - Complement factor H-related protein, tetranectin, β-2-microglobulin, and apolipoprotein C-II up-regulated and down-regulated after the Mediterranean diet and the low-fat diet, respectively. |
| Macnaughtan et al. (57) | Randomized, controlled, double blind, 2-arm parallel, 6-month intervention [multi-centre; original study] | Patients with cirrhosis (Child–Pugh score ≤10), aged 18-78 years (n = 87)  Sex: F = 25, M = 62 Age: 57.15±8.83 y Aetiology of Cirrhosis:  - Alcohol: n = 45 - Non-Alcohol (Hepatitis B or C, NASH, other): n = 42   Probiotic (n = 44) Sex: F = 12, M = 32 Age: 56.16±8.47 y Aetiology of Cirrhosis:  - Alcohol: n=21 - Non-Alcohol: n=23   Placebo (n = 43) Sex: F = 13, M = 30 Age: 58.16±9.18 y  Aetiology of Cirrhosis:  - Alcohol: n = 24 - Non-Alcohol: n = 19 | **Supplementation-based: Probiotic supplementation**  *Lactobacillus casei Shirota* (LcS,6.5x109 colony forming units (CFU)/bottle, 65 mL bottle 3 times/d for 6 months). | **Placebo administration**   (of similar-looking and tasting drink without bacteria, 65 mL bottle 3 times/d for 6 months). | **Phenotype-based stratification:**  **baseline stratification**   Stratification based on **aetiology of cirrhosis**: · **alcoholic cirrhotic patient** (n = 45) - LcS (n = 21) - Placebo (n = 24); · **non-alcoholic cirrhotic patient** (n = 42) - LcS (n = 23) - Placebo (n = 19). | Bio-Plex Pro human cytokine assay kit (Bio-Rad Laboratories Ltd., Watford, UK). [Targeted] | IL-1, IL-2, IL-4, IL-6, IL-8, IL-10, IL-12, IL-17A, IFN, MCP-1, MIP-1, TNFα [plasma]. | LcS intervention: - ↓ MCP-1 in the total population after 6 months (*p* = 0.02).  - No significant differences in IL-1β, IL-2, IL-4, IL-6, IL-8, IL-10, IL-1 p70, IL-17A, IFN, MIP-1β and TNFα at baseline, month 1 and month 6.   **Alcoholic patient cohort:** - ↓IL-1β (*p* = 0.04) at 6 months - ↓MCP-1 (*p* = 0.04) at 6 months. **Non-alcoholic patient cohort:** - ↓IL-17A (*p* = 0.02) at 6 months - ↓MIP-1β (*p* = 0.04) at 6 months. |
| Paganini et al. (58) | Randomized, controlled, double blind, 3-arm parallel, 4 month-intervention [single-centre; secondary analysis] | Healthy Kenyan infants aged 6.5–9.5 months (n = 75)   Sex: F = 41, M = 34 Age: 7.2 (7.0–8.2) m  Secretor (n = 54) Sex: F = 29, M = 25 Age: 7.2 (7.0–8.2) m Non-secretor (n = 21) Sex: F = 12, M = 9 Age: 7.1 (7.0–7.9) m   **Age reported as [median (IQR), months]* | **Supplementation-based:  Iron   - Fe group** (micronutrient powder (MNP, containing several minerals and vitamins, + 2.5 mg NaFeEDTA + 2.5 mg ferrous fumarate, daily for 4 months);  **- FeGOS group** (MNP as the Fe group, except maltodextrin was replaced with 10.5 g of 75% GOS daily for 4 months). | **Control group** (MNP containing several minerals and vitamins, but no iron and 10.5 g of maltodextrin, daily for 4 months). | **Phenotype-based stratification:  baseline stratification**  Stratification based on **maternal secretor status of breast milk HMO**: - **Secretor** (n = 54); - **Non-secreto**r (n = 21). | Multiplex immunoassay (not specified) [Targeted]. | PF, sTfR, CRP, AGP [plasma]. | Borderline significant time-by-secretor-status effect on PF (p = 0.052)  Significant: - time-by-intervention-group effect on sTfR (p = 0.002) - secretor-status-by-intervention-group effect on sTfR (p = 0.041) - time-by-intervention-group effect on BIS (p = 0.011) and total BIS (p = 0.003) - time-by-secretor-status effect on BIS (p = 0.023) and total BIS (p = 0.016).   Post hoc tests from baseline to 4 months: ↑ in PF (p = 0.043), BIS (p= 0.012) and total BIS (p=0.002) in the FeGOS-NS group. |
| Rodríguez-Cruz et al. (59) | Randomized, controlled, double blind, 2-arm parallel, 6-month intervention [multi-centre; original study] | Boys with a deletion in the Duchenne Muscular Dystrophy (DMD) gene or its promoter (detected by MPCR) aged between 3 and 18 years.  (n = 36)  ω-3 PUFA (n = 17) Age: 7.28 ± 3.08 y BMI: 38.38 (4.97, 96.7) Wheelchair-bound: n = 2 Placebo (n = 19) Age: 8.56 ± 3.08 y BMI: 70.86 (1.83, 99.4) Wheelchair-bound: n = 6  **BMI reported as percentile [median (min, max)]* | **Supplementation-based: ω-3 PUFA**  (10 capsules/d =2.9 g/day for 6 months). | **Placebo administration**   (10 identical placebo capsules/d containing sunflower oil blend for 6 months). | **Genotype-based stratification:  inclusion criterion**  Total population composed by individuals with a **deletion in the DMD gene or its promoter.** | Immunoassay kit of high sensitivity to human cytokine (Milliplex Map Immunoassay kit; Merck, Millipore, Billerica, MA, USA). [Targeted]. | IL-1β, IL-6, IL-10, TNF-α   [serum]. | ω-3 supplementation - Reduction trend (*p* = 0.067) of IL-1β at month 3 - ↑ IL-10 at months 3 (*p* = 0.012) and 6 (*p* = <0.005) - ↓IL-1β (p = 0.011) at month 6;  - ↓IL-6 (p = 0.041) at month 6;  - No changes in TNF-α during the 6 months. |
| Korpela et al. (60) | Randomized, controlled, double blind, 2-arm parallel, 6-month intervention [single-centre; secondary analysis] | 3 month-aged children from mothers whose fetuses were at high risk for allergy (n = 428)  Probiotic group (n = 199)* · breastfed (n = 168)  · formula-fed (n = 31)  Control group (n = 199)* · breastfed (n = 201)  · formula-fed (n = 22)   **excluding six infants with insufficient sequencing reads (< 100 reads)* | **Supplementation-based: Probiotic**   - **Mothers (**two capsules/d containing *Bifidobacterium breve Bb99*, *Propionibacterium freundenreichii subsp. shermanii JS*, *Lactobacillus rhamnosus Lc705* , L*actobacillus rhamnosus GG* , from 36 weeks of gestation until birth);    - **Infants (**same probiotic capsules once daily during the 6 months from birth). | **Placebo administration** - **Mothers** (two capsules/d with microcrystalline cellulose, from 36 weeks of gestation until birth);  - **Infants** (same control capsules once daily during the 6 months from birth) | **Phenotype-based stratification: baseline stratification**  Stratification based on **mode of delivery**:  · **Vaginally born infants** (n = 24) - control infants (n = 11) - supplemented infants (n = 13); · **Caesarean-born infants** (n = 24) - control infants (n = 12) - supplemented infants (n = 12). | LC-MS/MS on a nanoflow HPLC system (Easy-nLCII, Thermo Fisher Scientific) coupled to a LTQ Orbitrap Elite mass spectrometer (Thermo Fisher Scientific, Bremen, Germany) equipped with a nano-electrospray ionization source. * [Untargeted] ** Proteomic analysis employed in a subset of the cohort (n=48). All were fully breastfed and had received no antibiotic treatments.* | Metaproteome, not specified [feces]. | Bacterial metaproteomes differed between birth modes in the control group but were similar between birth modes in the supplemented group.  the supplemented groups (both birth modes) compared to the vaginally born control group showed a high level of induction (up to 50-fold) of **beta-galactosidase** and **beta-galactosyl *N*-acetyl hexosaminephosphorylase** (LNBP), bifidobacterial enzymes.  Bacteria in the caesarean-born infants expressed comparatively higher levels of **aspartate aminotransferase** and **aspartate ammonia lyase**  Metaproteome data were also used to predict the taxonomic origin of the proteins, and the obtained results appeared similar to those from the 16S rRNA gene data. |
| Ali et al. (61) | Randomized, controlled, triple blind, 2-arm parallel, 4-month intervention  [single-centre; original study] | Adults aged 18–75 years meeting Rome III criteria for any subtype of IBS with active symptoms (IBS SSS ≥150).. (n = 58)  Intervention (n = 29) Sex: F = 28, M = 1 Age: 38±13 y  BMI: 25.90±6.26 kg/m^2^ Comparison (n = 29) Sex: F = 27, M = 2 Age: 38±15 y BMI: 24.84±5.09 kg/m^2^ | **Dietary intervention: Individualized diet consistent with Leucocyte activation test results**  (for 4 weeks) | **Dietary intervention: Individualized sham diet systematically inconsistent with Leucocyte activation test results**  (for 4 weeks) | **Phenotype-based stratification: baseline stratification**   Individualized diet based on **leukocyte activation test,** | SomaLogic (Boulder, Colorado, USA) * [Targeted] **Proteomic analysis employed only on Strong responders (n=12):* | 1128 proteins, not specified [plasma]. | In paired analysis of the 12 plasma samples, concentrations of 87 of the 1128 proteins were significantly different (p<0.05) between baseline and week 4 samples.   After Benjamini-Hochberg correction: a significant pre–post difference in a single protein, **neutrophil elastase**, was found to be reduced in strong responders. |
| Waldvogel et al. (62) | Randomized controlled, double blind, 2-arm parallel, 4-week intervention [single-centre; secondary analysis] | Women donors, aged 18–50 years, presenting iron deﬁciency without anaemia one week after their blood donation). (n = 139)  Intervention (n = 69) Age: 33.4±8.3 y  Hepcidin: 0.16±0.14 nM Placebo (n = 70) Age: 30.7±8.8 y Hepcidin: 0.16±0.16 nM | **Supplementation-based: Iron** (80 mg/d oral ferrous sulphate for 4 weeks). | **Placebo administration**  (placebo pills for 4 weeks). | **Phenotype-based stratification: exploratory analysis**   Stratification based on **baseline hepcidin levels**: - Hepcidin: ≤ 0.11 nM  - Hepcidin: > 0.11 N21nM;  Stratification based on **post intervention** **ferritin increase**: - **Responder**: ferritin >30 ng/ml - **Non-responder**: ferritin ≤30 ng/ml;  Stratification based on **post intervention** **Hb increase**: - **Responder**: Hb ≥10 g/L - **Non-responder**: Hb <10 g/L. | Exactive Plus® Orbitrap LC-HRMS system (Thermo Scientific, Germany). [Targeted] | Hepcidin  [plasma]. | Baseline hepcidin stratification Hepcidin concentrations before supplementation did not modify the effects of supplementation on ferritin (p = 0.783) or haemoglobin (p= 0.322) concentrations 4 weeks later.   Post-intervention stratification  **Ferritin** ↑Hepcidin in **responders** vs non-responders (p=0.008) at 4 weeks;  **Hb** Similar Hepcidin concentrations between responders and non-responders to iron for haemoglobin (p = 0.477). |
| Chase et al. (63) | Randomized, controlled, double blind, 2-arm parallel, 36-month intervention [multi-center; secondary analysis] | Infants with HLA DR3 and/or DR4 alleles (n = 91) or a multiple first-degree relative with T1D (n = 7).    Group A (n = 41): infants whose mothers were enrolled during the third trimester of pregnancy.  Sex: F = 54%, M = 46% Breast-fed: n = 29 Weight: 3.6±0.7 kg  Group B (n = 57): infants enrolled within the first 5 months of life.  Sex: F = 49%, M = 51% Breast-fed: n = 39 Weight: 3.5±0.7 kg | **Supplementation-based: DHA supplementation**    - **nursing mothers** (800 mg DHA/d); -**babies** (10.2 mg DHA/oz or two 200 mg DHA capsules/d depending on age). | **Placebo administration**   - **nursing mothers** ( 800 mg corn/soy oil/d; - **babies** (3.4 mg DHA/oz or two 200-mg capsules/ day of control oil depending on age).     . | **Genotype-based stratification: inclusion criterion** Population composed by infants with HLA DR3 and/or DR4 alleles.   **(7 out of 98 were included based on phenotype at risk).* | Luminex multiplex assay (not specified)  [Targeted]. | IL-1ß, IL-6, IL-12p40, TNFα  [supernatants of diluted whole blood cultured with 1 μg/mL of LPS]. | - No significant differences between cytokine production in the control vs treatment infants at any of the 6 time periods; - Reduction of IL-1ß for all DHA-treated infants vs the breast-fed control infants at 12 months; - ↓ IL-1ß, IL-12p40, TNFα (p<0.05) in the DHA-treated infants at age 18 months; - No greater reduction at any time in inflammatory cytokine production in Group A vs Group B (data not shown).  After regression analysis:  - ↓ IL-12p40 at 30 months (p = 0.02) between control and treatment groups; - ↓ IL-1ß at 36 months (p = 0.04) between control and treatment groups; - No consistent differences in IL-6 and IL-10 between groups (data not shown). |
| De Luis et al. (64) | Randomized, controlled, 2-arm parallel, 9-month intervention [single-centre; original study] | Obese (BMI>30) non-diabetic individuals. (n = 193)  wild type genotype  Ala54/Ala54 (n = 99, 51.3%) Sex: F = 64.6%, M = 35.4% Age: 52.1±10.3y mutant type genotype Ala54/Thr54 (n = 80); Thr54/Thr54 (n = 14,); Sex: F = 75.5%, M = 24.5% Age:52.7 ± 11.4 y  HP diet (n = 99) - Wild type (n = 46),  BMI: 35.8±5.1kg/m^2^  - Mutant type (n = 53),  BMI: 36.7±4.1kg/m^2^  S diet (n = 94) - Wild type (n = 53), BMI:35.9±4.3 kg/m^2^ - Mutant type (n = 41), BMI:36.1±4.3 kg/m^2^ | **Dietary intervention:  High protein-low carbohydrate hypocaloric diet (HP diet)**  (for 9 months). | **Dietary intervention: Standard protein hypocaloric diet (S diet)**  (for 9 months). | **Genotype-based stratification: baseline stratification**   Stratification based on **genotype of FAPP2 polymorphism:** - **Wild type**: Ala54/Ala54 (n=99); - **Mutant type**: Ala54/Thr54 (n=80) or Thr54/Thr54 (n=14). | Multiplex Biorad© 10 plex assay (Bio-Rad®, Hercules, Calif., USA). [Targeted] | Resistin, leptin, adiponectin [plasma]. | HP diet Decrease in leptin (p > 0.05) in both genotypes. S diet Decrease in leptin (p > 0.05) in both genotypes.   The amount of leptin decrease was similar with both diets.  No differences were detected among basal and post-treatment values of adipocytokines among genotypes.  Resistin and adiponectin levels remained unchanged along 9 months. |
| Heng et al. (65) | Randomized, controlled, 2-arm parallel, 6 month-intervention  [single-centre; original study] | Malays females aged 33-52 years. (n = 14)  young group (n = 6) Age: 34.6±0.8 y BMI: 25.9±3.2 kg/m^2^ Intervention: n = 3 Placebo: n = 3 old group (n = 8) Age: 49.5±0.9 y BMI: 26.0±1.6 kg/m^2^ Intervention: n = 4 Placebo: n = 4 | **Supplementation-based: Tocotrienol-rich fraction (TRF)**   (150 mg/day for 6 months). | **Placebo administration**  (placebo capsules for 6 months). | **Phenotype-based stratification: baseline stratification**   Stratification based on **age at baseline**: - **Young group**: 32±2 y; - **Old group**: 52±2 y. | MALDI-TOF/TOF mass spectrometer (ABI 4800 plus, Applied Biosystems) in the positive ion reflector mode. [Untargeted] | No specific protein  [plasma]. | After TRF supplementation: Significant change in expression for 12 protein spots (*P* < 0.05) which represented 8 proteins, classified into 4 categories: - cholesterol homeostasis: APOA, APOE;  - acute-phase response: CRP, HTPR; - protease inhibitor: CBPN, AMBP;  - immune response: FHR1, FHR2.   In both groups, changes in protein expression were first observed after 3 months.  - **Young group**: 3 proteins (APOE, FHR2, and AMBP) upregulated after 3 months.  - **Old group**: 6 proteins (CBPN, FHR1, HPTR, APOE, FHR2, and AMBP) upregulated after 3months.   The remaining regulated proteins only changed in expression after 6 months: all proteins were upregulated except for CRP, which was downregulated. |
| Barona et al. (66) | Randomized, controlled, double blind, cross-over, two 4-week interventions separated by 3-week washout phase [not specified; secondary analysis] | Men aged 30–70 years classified with MetS (n = 24)  Men with dyslipidaemia (n = 11)  Age: 48.1±11.3 y  Waistline: 107.4±10.5 cm  Men without dyslipidaemia  (n = 13)  Age: 53.9±7.4 y  Waistline: 109.2±16.1 cm | **Supplementation-based: Freeze-dried whole grape powder (GRAPE**  (46 g (= 2 cups or 252 g of grapes) for 4weeks). | **Placebo administration**  (46 g of placebo matching macronutrient composition and characteristics of GRAPE, except for the polyphenols, for 4weeks). | **Phenotype-based stratification: baseline stratification**    Stratification based on **MetS subsets**: - **Dyslipidemic subjects**:  - **Non-dyslipidemic subjects**: | Human CVD Panel 3 MILLIPLEX® MAP kit; Human CVD Panel 1 MILLIPLEX®MAP kit (Millipore Corporation, Billerica, MA, USA); based on Luminex® xMAP® technology (Austin, TX, USA). [Targeted] | TNF-α, IL-6, IL-8, IL-10; Adiponectin [plasma]. | [Plasma adiponectin (p<0.005) and IL-10 (p<0.05) had opposite responses based on dyslipidaemia category: **- men with dyslipidaemia**: ↓ adiponectin, ↓ IL-10; **- men without dyslipidaemia**: ↑ adiponectin, ↑IL-10.  IL-6, IL-8, and TNF-α did not differ between periods regardless of dyslipidaemia classification.](https://www.mdpi.com/2072-6643/4/12/1945#table_body_display_nutrients-04-01945-t002) |
| Rubio‐Aliaga et al. (67) | Randomized, controlled, 5-arm parallel, 26-week intervention [multi-centre; secondary analysis]  *For this secondary analysis were considered just the Group 1 and 2* | Subset of overweight/obese, non-diabetic women from the DiOGenes cohort (n = 24)  Group 1: LP/LGI (n = 12) - Weigh loser BMI: 86.55±11.63 kg/m^2^ - Weigh re-gainer BMI:85.4±15.78 kg/m^2^  Group 2: LP/HGI (n = 12) - Weigh loser BMI: 90.95±22.72 kg/m^2^ - Weigh re-gainer BMI: 88.78±6.94 kg/m^2^ | **Dietary intervention:**  **Low calorie diet** (**LCD**) for 8 weeks  +  **Low protein (LP)/low glycaemic index (LGI) diet** (for 6 months) | **Dietary intervention:**  **LCD** for 8 weeks +  **LP/high glycaemic index (HGI) diet** (for 6 months). | **Phenotype-based stratification: exploratory analysis**   Stratification based on **weight maintenance success**: - **Weight loser** (successful, n = 6 for each diet); - **Weight re-gainer** (unsuccessful, n = 6 for each diet). | NanoLC-ESI-MS/MS system, HCT ultra ion-trap MS (Bruker Daltonics, Bremen, Germany) coupled on-line to an Ultimate 3000 HPLC system (Dionex, Olten, Switzerland) equipped with an analytical Magic C reversed-phase column (100×0.075 mm, 5 μm).  [Untargeted] | No specific protein [plasma]. | Total protein identified: n= 75   18 proteins differentially expressed (p<0.1) as a function of success rate and protein content in the LGI diet: - LP/LGI diet: **APOE, ITIH1, ORM1, PON1, PROS1, PZPZ;** - HP/LGI diet: **C8A, C9, KNG1, PROS1, PZP, RBP4, SERPINF1.** 6 proteins were candidate biomarkers to distinguish HP vs LP diet within the weight loser group, and 3 within the weight re-gainer group. - Reduction in PROS1 and increase in PZP were indicative of successful weight maintenance and also discriminated HP vs LP diet among successful individuals;  - PROS1 altered depending on both success and diet;  - C4BPA did not show altered plasma levels in any condition.  - PZP was significantly different between most conditions and showed the opposite profile compared with PROS1.  Protein network analysis (HP/LGI diet): success rate for HP dietary content is indicated by 4 down-regulated (PZP, C9, CRP and APCS) and 7 up-regulated proteins (APOM, APOL1, RBP4, IGFALS, ORM1, ADIPOQ and PROS1). |
| Brauer et al. (68) | EAT study: Randomized, controlled, cross-over, four 6-day interventions separated by a 14-day washout phase [single-centre; secondary analysis];  2EAT study: Randomized, controlled, cross-over, four 6-day intervention separated by at least a 21-day washout phase [single-centre; secondary analysis] | EAT study: Healthypartecipants aged 20-40 years (n = 36)  - GSTM1+:  Sex: F = 4; M = 10 - GSTM1-:  Sex: F = 13; M = 9  EAT2 study: Healthypartecipants aged 20-40 years (n = 42)  - GSTM1+:  Sex: F = 10; M = 10 - GSTM1-:  Sex: F = 7; M = 15 | **Food-based intervention: cruciferous vegetables**   EAT study: (**basal diet + cruciferous vegetables** (436 g/day);for 6 days)  2EAT study: (**basal diet supplemented with cruciferous vegetables**  (7 g/kg body weight);   **basal diet supplemented with twice the amount of cruciferous vegetables** (14 g/kg body weight); each diet lasted 14 days with at least a 21 day washout period between diets). | **Control group** (basal diet devoid of fruit and vegetables). | **Genotype-based stratification: baseline stratification**   Stratification based on **GSTM1 genotype**: - **GSTM1- genotype** - EAT (n = 14) - 2EAT (n = 20); - **GSTM1+ genotype** - EAT (n = 22) - 2EAT (n = 22). | MALDI-TOF MS on an Applied Biosystem Voyager DE-Pro spectrometer (Foster City, CA) + nano LC/MS using a ThermoFisher LTQ (Thermo Fisher Scientific, Waltham, MA).  [Untargeted] | No specific protein [serum]. | EAT study: 24 peaks differed between the two diets (p < 0.05). **Stratifying by genotype**: 21 peaks from GSTM1-null and 39 peaks from GSTM1+ individuals changed significantly. Of the original 24 peaks, 20 remained significant in at least one genotype. 2EAT study (2X cruciferous diet): Twenty-four peaks differed from the basal diet (p < 0.05) **Stratifying 2X data by genotype**: nine-teen of twenty-four peaks remained significant in at least one genotype. In addition, fifteen additional peaks were unique to GSTM1-null individuals, and twenty-three peaks were unique to GSTM1+ individuals.  Combined analysis of diet and genotype in EAT and 2EAT **- genotype-independent peaks**: 4 peaks shared significant changes and maintained the same trend and intensity change in both sample populations.  **- genotype-independent peaks**: 2 additional peaks (6500 and 6700 m/z) changed consistently.  Among the six key peaks, 4905 and 9812 m/z increased, while 4813, 6500, 6700, and 9565 m/z decreased in response to cruciferous vegetable intake.  Twopeaks were selected for identification: - 6700 m/z peak: a fragment of TTR, ↓ with cruciferous intake, with a larger reduction among GSTM1+ vs GSTM1-null (e.g., in 2EAT: p = 0.001). Comparison between genotypes was significant (p < 0.001) for cruciferous intake across both studies.   - 9565 m/z peak: a fragment of ZAG, ↓ with cruciferous intake in GSTM1+ individuals compared with the basal diet (p < 0.001). The difference between genotypes was significant across all cruciferous diets in both studies (p < 0.001). |
| Lehtonen et al. (69) | Randomized, controlled, crossover, two 33/35-day interventions separated by a 30/39-day washout phase [single-centre; original study] | Overweight/obese women (n = 80)  Age: 44.2±6.2 y BMI: 29.6±2.1 kg/m^2^ | **Supplementation-based: Berries**   (**Bilberries** (BB), **Sea buckthorn** (SB,) **SB phenolic extract** (SBe), **SB oil** (Sbo);  equivalent to 100 g fresh berries for 33–35 days). | Each berry intervention served as a comparator to the others | **Phenotype-based stratification: exploratory analysis**   Stratification based on baseline **BMI quartiles**:  - Q1: 26.0–27.7 kg/m2;  - Q2: 27.7–29.5 kg/m2;  - Q3: 29.5–31.2 kg/m2;  - Q4: 31.2–39.7 kg/m2. | Millipore's Human CVD1-kit (HCVD1-67AK) (Millipore, Billerica, MA, USA) using a Bio-Rad Bio-Plex 200 System (Bio-Rad Laboratories, Espoo, Finland).  [Targeted] | sICAM-1, sVCAM-1, adiponectin;  [plasma]. | Decrease in VCAM concentrations was higher in subjects with higher BMI after SBo intervention (not specified). - **BMI 26.6–27.7**: no changes in VCAM  - **BMI 29.5–31.2**: highest reduction in VCAM   BB diet: no different change in VCAM, except in the fourth quartile **BMI 31.2–39.7** where the change was minor;  SB diet: only **BMI 26–27.7** had reduction in VCAM values; SBe diet**:** highest reduction in VCAM for **BMI 31.2–39.7** |
| Kim et al. (70) | Randomized, controlled, double blind, 3-arm parallel, 8-week intervention [single-centre; original study] | Healthy men aged 22–57 years, who frequently smoked cigarettes or consumed alcohol. (n = 116)  low dose lycopene (n = 41) Age: 34.8±1.28 y  BMI: 25.3±0.60 kg/m2 high dose lycopene (n = 37) Age: 34.7±1.23 y BMI: 23.9±0.49 kg/m2 placebo (n = 38) Age: 33.5±1.13 y  BMI: 24.9±0.50 kg/m2 | **Supplementation-based: Lycopene   (Low dose:** one capsule/d of 6 mg for 8 weeks;  **High dose**: one capsule/d of 15 mg for 8 weeks). | **Placebo administration**  (one capsule/d of 6-mg soybean oil for 8 weeks). | **Phenotype-based stratification:**  **exploratory analysis**  Stratification based on **baseline levels of markers for endothelial cell function**:  - **Impaired endothelial cell functions:** baseline level of at least one of these markers was upper tertile (sVCAM-1 or sICAM-1) or lower tertile (RH-PAT) (n = 76); - **Normal function:** any other case (n = 40). | Bio-Plex™ Reagent Kit with the Bio-Plex™ (Bio-Rad Laboratories, Inc., Hercules, CA, USA).  [Targeted] | sVCAM-1, sICAM-1  [plasma]. | **Subjects with** **impaired endothelial cell functions**:  - No significant differences in initial levels among three groups. - Reduction in sICAM-1 in both low-dose and high-dose supplementation compared vs placebo. The reduction is greater in the high-dose group, but not significant. - Reduction in sVCAM-1 in both treatment group, ↑ (p=0.048) in the high-dose group vs placebo.  No significant inter-group differences in subjects with normal endothelial cell function (data not shown). |
